# Squidpy: a scalable framework for spatial omics analysis

**DOI:** 10.1038/s41592-021-01358-2

**Published:** 2022-01-31

**Authors:** Giovanni Palla, Hannah Spitzer, Michal Klein, David Fischer, Anna Christina Schaar, Louis Benedikt Kuemmerle, Sergei Rybakov, Ignacio L. Ibarra, Olle Holmberg, Isaac Virshup, Mohammad Lotfollahi, Sabrina Richter, Fabian J. Theis

**Affiliations:** 1 Institute of Computational Biology, Helmholtz Center Munich, Munich, Germany; 2 TUM School of Life Sciences Weihenstephan, Technical University of Munich, Munich, Germany; 3 Institute for Tissue Engineering and Regenerative Medicine (iTERM), Helmholtz Center Munich, Munich, Germany; 4 Department of Mathematics, Technical University of Munich, Munich, Germany; 5 Department of Anatomy and Physiology, University of Melbourne, Melbourne, Victoria Australia

**Keywords:** Software, Transcriptomics, Data integration, Imaging

## Abstract

Spatial omics data are advancing the study of tissue organization and cellular communication at an unprecedented scale. Flexible tools are required to store, integrate and visualize the large diversity of spatial omics data. Here, we present Squidpy, a Python framework that brings together tools from omics and image analysis to enable scalable description of spatial molecular data, such as transcriptome or multivariate proteins. Squidpy provides efficient infrastructure and numerous analysis methods that allow to efficiently store, manipulate and interactively visualize spatial omics data. Squidpy is extensible and can be interfaced with a variety of already existing libraries for the scalable analysis of spatial omics data.

## Abstract (web summary)

Squidpy enables comprehensive analysis and visualization of spatial omics data and image with high efficiency.

## Main

Dissociation-based single-cell technologies have enabled the deep characterization of cellular states and the creation of cell atlases of many organs and species^1^. However, how cellular diversity constitutes tissue organization and function is still an open question. Spatially resolved molecular technologies aim at bridging this gap by enabling the investigation of tissues in situ at cellular and subcellular resolution^2–4^. In contrast to the current state-of-the-art dissociation-based protocols, spatial molecular technologies acquire data in greatly diverse forms, in terms of resolution (few cells per observation to subcellular resolution), multiplexing (dozens of features to genome-wide expression profiles), modality (transcriptomics, proteomics and metabolomics) and often with an associated high-content image of the captured tissue^2–4^. Such diversity in generated data and corresponding formats currently represents an infrastructural hurdle that has hampered urgently needed development of interoperable analysis methods. The underlying computational challenges lie in efficient data representation as well as comprehensive analysis and visualization methods.

Existing analysis frameworks for spatial data focus either on preprocessing^5–8^ or on one particular aspect of spatial data analysis^9–13^. The combination of different analysis steps is still hampered by the lack of a unified data representation and of a modular application programming interface, for example loading processed data from Starfish^5^, combining stLearn’s^11^ integrative analysis of tissue images together with Giotto’s powerful spatial statistics^13^, BayesSpace spatial clustering^14^ or leveraging state-of-the-art deep-learning-based methods for image segmentation^15,16^ and visualization^17^. A comprehensive framework that enables community-driven scalable analyses of both spatial neighborhood graph and image, along with an interactive visualization module, is missing (Supplementary Table 1).

**Fig. 1:**
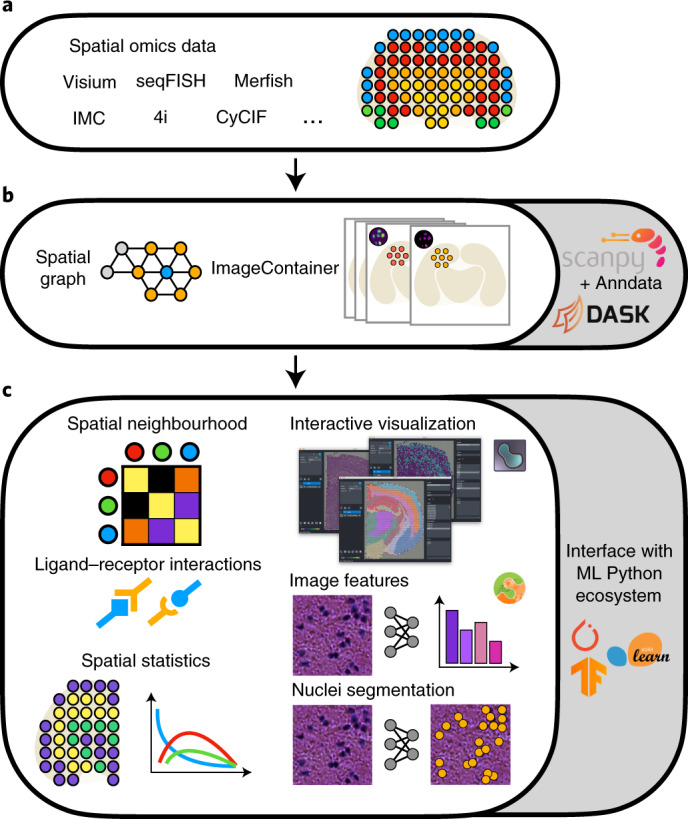
Squidpy is a software framework for the analysis of spatial omics data. **a**, Squidpy supports inputs from diverse spatial molecular technologies with spot-based, single-cell or subcellular spatial resolution. **b**, Building upon the single-cell analysis software Scanpy^20^ and the Anndata format, Squidpy provides efficient data representations of these inputs, storing spatial distances between observations in a spatial graph and providing an efficient image representation for high-resolution tissue images that might be obtained together with the molecular data. **c**, Using these representations, several analysis functions are defined to quantitatively describe tissue organization at the cellular (spatial neighborhood) and gene level (spatial statistics, spatially variable genes and ligand–receptor interactions), to combine microscopy image information (image features and nuclei segmentation) with omics information and to interactively visualize high-resolution images.

For this purpose we developed ‘Spatial Quantification of Molecular Data in Python’ (Squidpy), a Python-based framework for the analysis of spatially resolved omics data (Fig. 1). Squidpy aims to bring the diversity of spatial data in a common data representation and provide a common set of analysis and interactive visualization tools. Squidpy introduces two main data representations to manage and store spatial omics data in a technology-agnostic way: a neighborhood graph from spatial coordinates and large-source tissue images acquired in spatial omics data (Fig. 1b). Both data representations leverage sparse^18^ or memory-efficient^19^ approaches in Python for scalability and ease of use. They are also able to deal with both two-dimensional and three-dimensional (3D) information, thus laying the foundations for comprehensive molecular maps of tissues and organs. Such infrastructure is coupled with a wealth of tools that enable the identification of spatial patterns in tissue and the mining and integration of morphology data from large tissue images (Fig. 1c). Squidpy is built on top of Scanpy and Anndata^20^ and it relies on several scientific computing libraries in Python, such as Scikit-image^21^, Napari^22^ and Dask^19^. Its modularity makes it suitable to be interfaced with a variety of additional tools in the Python data science and machine-learning ecosystem (such as external segmentation methods and modern deep-learning frameworks), as well as several single-cell data analysis packages. It provides a rich documentation, with tutorials and example workflows, integrated in the continuous integration pipeline. It allows users to quickly explore spatial datasets and lays the foundations for both spatial omics data analysis as well as development of new methods. Squidpy is available at https://github.com/theislab/squidpy; documentation and extensive tutorials covering the presented results and more are available at https://squidpy.readthedocs.io/en/latest/.

## Results

### Squidpy provides infrastructure and analysis tools to identify spatial patterns in tissue

**Fig. 2:**
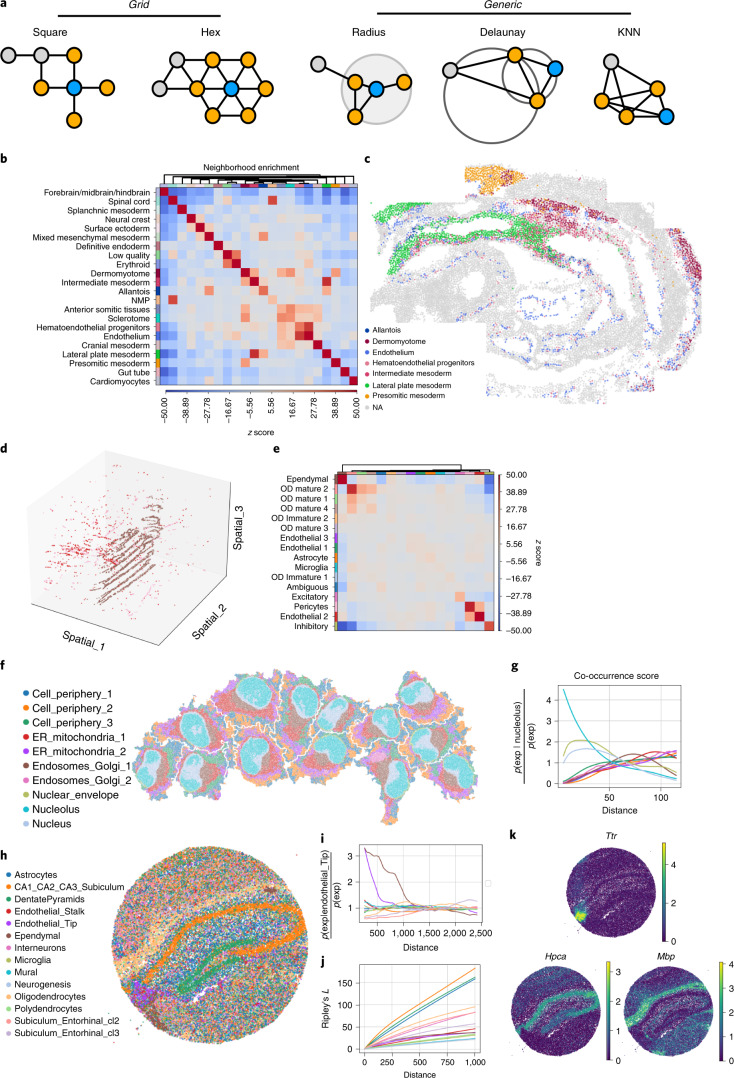
Analysis of spatial omics datasets across diverse experimental techniques using Squidpy. **a**, Example of nearest-neighbor graphs that can be built with Squidpy: grid-like and generic coordinates. **b**, Neighborhood enrichment analysis between cell clusters in spatial coordinates. Positive enrichment is found for the following cluster pairs: ‘Lateral plate mesoderm’ with ‘Allantois’ and ‘Intermediate mesoderm’ clusters, ‘Endothelium’ with ‘Hematoendothelial progenitors’, ‘Anterior somitic tissues’, ‘Sclerotome’ and ‘Cranial mesoderm’ clusters, ‘NMP’ with ‘Spinal cord’, ‘Allantois’ with ‘Mixed mesenchymal mesoderm’, ‘Erythroid’ with ‘Low quality’, ‘Presomitic mesoderm’ with ‘Dermomyotome’ and ‘Cardiomyocytes’ with ‘Mixed mesenchymal mesoderm’. These results were also reported by the original authors^33^. NMP, neuromesodermal progenitor. **c**, Visualization of selected clusters of the seqFISH mouse gastrulation dataset. **d**, Visualization in 3D coordinates of three selected clusters in the MERFISH dataset^34^. The ‘Pericytes’ are in pink, the ‘Endothelial 2’ are in red and the ‘Ependymal’ are in brown. The full dataset is visualized in Supplementary Fig. 2g. **e**, Results of the neighborhood enrichment analysis. The ‘Pericytes’ and ‘Endothelial 2’ clusters show a positive enrichment score. OD, oligodendrocytes. **f**, Visualization of subcellular molecular profiles in HeLa cells, plotted in spatial coordinates (approximately 270,000 observations/pixels). ER, endoplasmic reticulum. **g**, Cluster co-occurrence score computed for each cell, at increasing distance threshold across the tissue. The cluster ‘Nucleolus’ is found to be co-enriched at short distances with the ‘Nucleus’ and the ‘Nuclear envelope’ clusters. **h**, Visualization of SlideseqV2 dataset with cell-type annotations^35^. **i**, Cluster co-occurrence score computed for all clusters, conditioned on the presence of the ‘Ependymal’ cluster. At short distances, there is an increased colocalization between the ‘Endothelial_Tip’ cluster and the ‘Ependymal’ cluster. **j**, Ripley’s *L* statistics computed at increasing distances; clusters such as ‘CA1_CA2_CA3_Subiculum’ and ‘DentatePyramids’ show high Ripley’s *L* values across distances, providing quantitative evaluation of the ‘clustered’ spatial pattern across the slide. Clusters such as the ‘Endothelial_Stalk’, with a lower Ripley’s *L* value across increasing distances, have a more ‘random’ pattern. **k**, Expression of top three spatially variable genes (*Ttr*, *Mbp* and *Hpca*) as computed by Moran’s *I* spatial autocorrelation on the SlideseqV2 dataset. They seem to capture different patterning and specificity for cell types (‘Endothelial_Tip’, ‘Oligodendrocytes’ and ‘CA1_CA2_CA3_Subiculum’, respectively).

Spatial proximity is encoded in spatial graphs, which require flexibility to support the variety of neighborhood metrics that spatial data types and users may require. For instance, in Spatial Transcriptomics (ST^23^, Visium^24^ and DBit-seq^25^), a node is a spot and a neighborhood set can be defined by a fixed number of adjacent spots (square or hexagonal grid; Fig. 2a), whereas in imaging-based molecular data (seqFISH^26^, MERFISH^27^, Imaging Mass Cytometry^28,29^, CyCif^30^, 4i^31^ and Spatial Metabolomics^32^; Fig. 2a), a node can be defined as a cell (or pixel) and a neighborhood set can also be chosen based on a fixed radius (expressed in spatial units) from the centroid of each observation. Alternatively, other approaches, such as Euclidean distance or Delaunay triangulation, can be utilized to build the neighbor graph (Fig. 2a). Squidpy can compute all the aforementioned modalities thus making it technology-agnostic and providing the infrastructure for downstream analysis tools that aim at quantifying spatial organization of the tissue.

A key question in the analysis of spatial molecular data is the description and quantification of spatial patterns and cellular neighborhoods across the tissue. Squidpy provides several tools that leverage the spatial graph to address such questions. On a recently published seqFISH^33^ dataset we built a spatial nearest-neighbor graph based on Delaunay triangulation and computed a permutation-based neighborhood enrichment across cell-type annotations (Methods). Clusters of enriched cell types (such as ‘Lateral plate mesoderm’ with ‘Allantois’ and ‘Intermediate mesoderm’ clusters, ‘Endothelium’ with ‘Hematoendothelial progenitors’; Fig. 2b) are consistent with the original publication^33^ and the spatial proximity can be visualized in Fig. 2c. A similar analysis was performed on a MERFISH dataset^34^, where we could identify a neighborhood enrichment between ‘Endothelial 2’ and ‘Pericytes’ clusters, whereas the ‘Ependymal’ cluster shows a strong co-enrichment with itself but depleted enrichment with the other clusters (Fig. 2d,e shows selected clusters and Supplementary Fig. 2g shows the the full dataset). Furthermore, our implementation is scalable and ~tenfold faster than a similar implementation in Giotto^13^ (Supplementary Fig. 1a,b and Supplementary Table 2 show extensive comparisons), enabling analysis of large-scale spatial omics datasets. Sparse and scalable implementation in Squidpy enables working with subcellular-resolution spatial data such as 4i^31^. We considered ~270,000 pixels as subcellular resolution observations across 13 cells (Fig. 2f) and evaluated their cluster co-occurrence at increasing distances (Fig. 2g). As expected, the subcellular measurements annotated in the nucleus compartment co-occur together with the nucleus and the nuclear envelope, at short distances. The co-occurrence score represents an interpretable score to investigate patterns of spatial organization in tissue. When applied to a SlideseqV2 dataset^35^ (Fig. 2h), the co-occurrence score could provide a quantitative indication of a qualitative observation that the ‘Endothelial_Tip’ cluster shows a strong co-occurrence with the ‘Ependymal’ cluster (Fig. 2i). To obtain a global indication of the degree of clustering or dispersion of a cell-type annotation in the tissue area, the Ripley’s *L* can be computed. When applied to the same dataset (Fig. 2j), it highlighted how the ‘CA1 CA2 CA3 Subiculum’ and the ‘Dentate Pyramids’ annotations have a more ‘clustered’ spatial patterning than other annotations, such as the ‘Endothelial Stalk’. Squidpy implements three variations of the Ripley statistic (*L*, *F* and G; Supplementary Fig. 2b provides an additional example) that allows one to gain a global understanding of spatial patterning of discrete covariates. Finally, to identify genes that show strong spatial variability, we applied the Moran’s *I* spatial autocorrelation statistics (Methods) and visualized the three top genes (Fig. 2k; *Ttr*, *Mbp* and *Hpca*), which all show different spatial patterns and seem to largely colocalize with cell-type annotations (‘Endothelial Tip’, ‘Oligodendrocytes’ and ‘CA1 CA2 CA3 Subiculum’, respectively).

These statistics yield interpretable results across diverse experimental techniques, as demonstrated on an Imaging Mass Cytometry dataset^36^, where we showcase additional methods such as Ripley’s *F* function, average clustering and degree and closeness centrality (Supplementary Fig. 2). In conclusion, Squidpy provides a suite of orthogonal analysis tools that enable analysts to gain a quantitative understanding of the spatial organization of cellular and subcellular units.

### Squidpy enables analysis and visualization of large images in spatial omics data

**Fig. 3:**
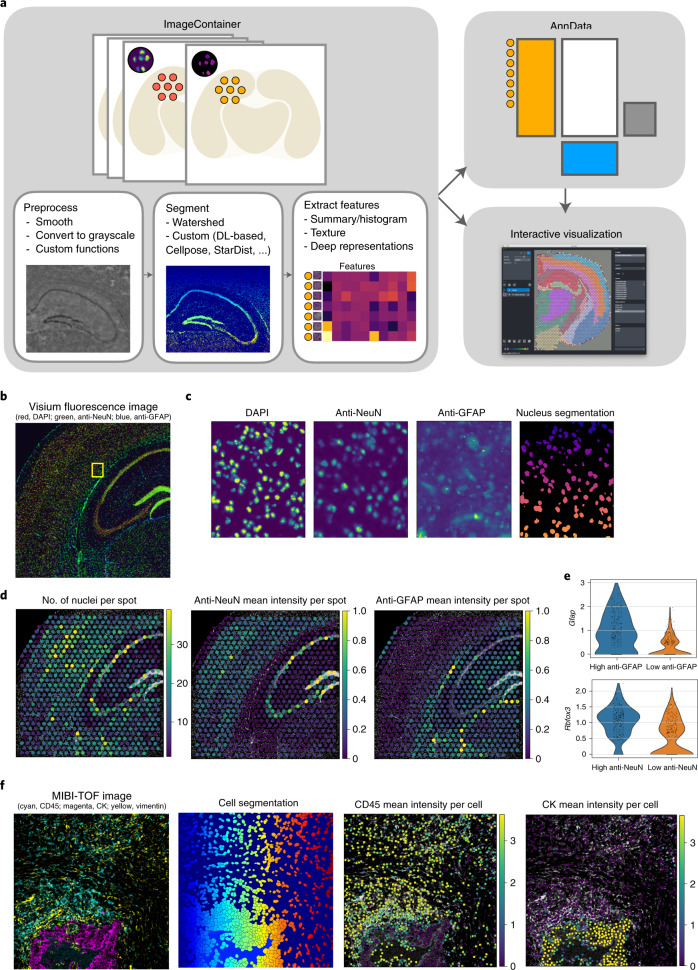
Image analysis and relating images to molecular profiles with Squidpy. **a**, Schematic drawing of the ImageContainer object and its relation to Anndata. The ImageContainer object stores multiple image layers with spatial dimensions *x*, *y*, *z* (left). An exemplary image-processing workflow consisting of preprocessing, segmentation and feature extraction is shown in the bottom. Using image features, pixel-level information is related to the molecular profile in Anndata (top right). Anndata and ImageContainer objects can be visualized interactively using napari (bottom right). DL, deep-learning. **b**, Fluorescence image with markers DAPI, anti-NeuN and anti-GFAP from a Visium mouse brain dataset (https://support.10xgenomics.com/spatial-gene-expression/datasets). The location of the inset in **c** is marked with a yellow box. **c**, Details of fluorescence image from **b**, showing from left to right the DAPI, anti-NeuN and anti-GFAP channels and nuclei segmentation of the DAPI stain using watershed segmentation. **d**, Image features per Visium spot computed from fluorescence image in **b**. From left to right are shown: number of nuclei in each Visium spot derived from the nuclei segmentation, the mean intensity of the anti-NeuN marker in each Visium spot and the mean intensity of the anti-GFAP marker in each Visium spot. **e**, Violin plot of log-normalized *Gfap* and *Rbfox3* gene expression in Visium spots with low and high anti-GFAP and anti-NeuN marker intensity (lower and higher than median marker intensity), respectively. **f**, Calculation of per-cell features from a MIBI-TOF dataset^41^. Tissue image showing three markers CD45, CK and vimentin (left). Cell segmentation provided by the authors^41^ (center left). Mean intensity of CD45 per cell derived from the raw image using Squidpy (center right). Mean intensity of CK per cell derived from the raw image using Squidpy (right). For quantitative comparison see Supplementary Fig. 2. This example is part of the Squidpy documentation (https://squidpy.readthedocs.io/en/latest/auto_tutorials/tutorial_visium_fluo.html and https://squidpy.readthedocs.io/en/latest/auto_tutorials/tutorial_mibitof.html).

The high-resolution microscopy image additionally captured by spatial omics technologies represents a rich source of morphological information that can provide key biological insights into tissue structure and cellular variation. Squidpy introduces a new data object, the ImageContainer, which efficiently stores the image with an on-disk/in-memory switch based on xArray and Dask^19,37^. This object provides a general mapping between pixel coordinates and molecular profiles, enabling analysts to relate image-level observations to omics measurements (Fig. 3a). It provides seamless integration with napari^22^, thus enabling interactive visualization of analysis results stored in an Anndata object alongside the high-resolution image directly from a Jupyter notebook. It also enables interactive manual cropping of tissue areas and automatic annotation of observations in Anndata. As napari is an image viewer in Python, all the above-mentioned functionalities can be also interactively executed without additional requirements. Following standard image-based profiling techniques^38^, Squidpy implements a pipeline based on Dask Image^19^ and Scikit-image^21^ for preprocessing and segmenting images, extracting morphological, texture and deep-learning-powered features (Fig. 3a). To enable efficient processing of very large images, this pipeline utilizes lazy loading, image tiling and multiprocessing (Supplementary Fig. 1b). When using image tiling during processing, overlapping crops are used to mitigate border effects. Features can be extracted from a raw-tissue image crop or Squidpy’s segmentation module can be used to extract segmentation objects (nuclei or cells) counts, sizes or general image features at segmentation-mask level (Supplementary Fig. 2b).

For segmentation, Squidpy provides a pipeline based on the watershed algorithm and provides an interface to state-of-the-art nuclei segmentation algorithms such as Cellpose^16^ and StarDist^15^ (Supplementary Fig. 5a). As an example for segmentation-based features, we computed nuclei segmentation using the 4,6-diamidino-2-phenylindole (DAPI) stain of a fluorescence mouse brain section (Fig. 3b,c) and showed the estimated number of nuclei per spot on the hippocampus (Fig. 3d, left). The cell-dense pyramidal layer can be easily distinguished with this view of the data, showcasing the richness and interpretability of information that can be extracted from tissue images when brought in a spot-based format. In addition, we can leverage segmented nuclei to inform cell-type deconvolution (or decomposition/mapping) methods such as Tangram^39^ or Cell2Location^40^. In Supplementary Fig. 4 we showcase how priors on nuclei densities derived from nuclei segmentation in Squidpy can be used both for inferring cell-type proportions as well as mapping cell types to segmentation objects with Tangram.

Image-based features contained in Squidpy include built-in summary, histogram and texture features and more-advanced features such as deep-learning-based (Supplementary Fig. 2a) or CellProfiler (Supplementary Fig. 5b) pipelines provided by external packages.

Using the anti-NeuN and anti-glial fibrillary acidic protein (GFAP) channels contained in the fluorescence mouse brain section, we calculated their mean intensity for each Visium spot using summary features (Fig. 3d center and right). This image-derived information relates well to molecular information: Visium spots with high marker intensity have a higher expression of *Rbfox3* (for anti-NeuN marker) and *Gfap* (for anti-GFAP marker) than low-marker-intensity spots (Fig. 3e). Image features can also be calculated at the spot level, thus aggregating several cells or at an individual per-cell level. Using a multiplexed ion beam imaging by time of flight (MIBI-TOF) dataset^41^ with a previously calculated cell segmentation, we calculate mean intensity features of two markers contained in the original image (Fig. 3f). The calculated mean intensities have a high correlation with the associated mean intensity values contained in the associated molecular profile (Supplementary Fig. 3a). These results highlight how explicitly analyzing image-level information leads to insightful validation but also potentially new hypotheses.

### Squidpy’s workflow enables the integrative analysis of spatial transcriptomics data

The feature extraction pipeline of Squidpy allows the comparison and joint analysis of spatial patterning of the tissue at the molecular and morphological level. Here, we show how Squidpy’s functionalities can be combined to analyze 10X Genomics Visium spatial transcriptomics data of a coronal mouse brain section.

**Fig. 4:**
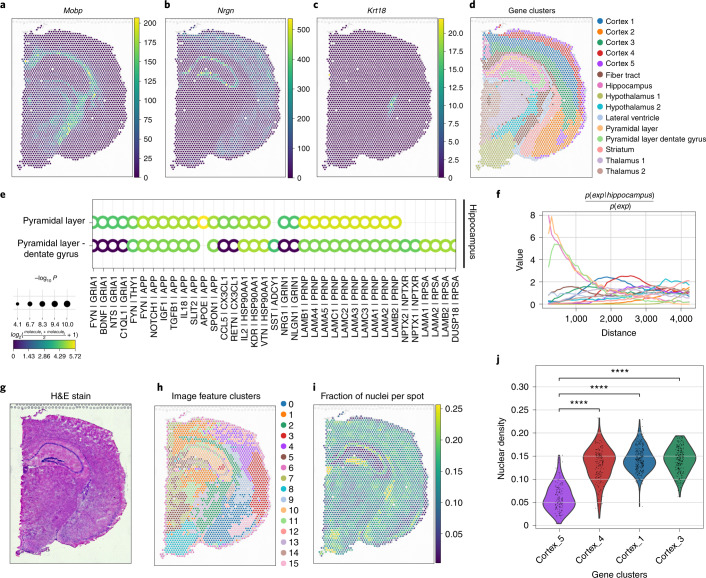
Analysis of mouse brain Visium dataset using Squidpy. **a**,**b**, Gene expression in spatial context of two spatially variable genes (*Mobp* and Nrgn) as identified by Moran’s *I* spatial autocorrelation statistic. **c**, Gene expression in spatial context of one spatially variable gene (*Krt18*) identified by the Sepal method^42^. **d**, Clustering of gene expression data plotted on spatial coordinates. **e**, Ligand–receptor interactions from the cluster ‘Hippocampus’ to clusters ‘Pyramidal layer’ and ‘Pyramidal layer dentate gyrus’. Shown are a subset of significant ligand–receptor pairs queried using the Omnipath database. Shown ligand–receptor pairs were filtered for visualization purposes, based on expression (mean expression > 13) and significant after false discovery rate (FDR) correction (*P* < 0.01). *P* values results from a permutation-based test with 1,000 permutations and were adjusted with the Benjamini–Hochberg method. **f**, Co-occurrence score between ‘Hippocampus’ and the rest of the clusters. As seen qualitatively by clusters in a spatial context in **d**, ‘Pyramidal layer’ and ‘Pyramidal layer dentate gyrus’ co-occur with the Hippocampus at short distances, given their proximity. **g**, H&E stain. **h**, Clustering of summary image features (channel intensity mean, s.d. and 0.1, 0.5, 0.9th quantiles) derived from the H&E stain at each spot location (for quantitative comparison to gene clusters from **d** see Supplementary Fig. 2e). **i**, Fraction of nuclei per Visium spot, computed using the cell segmentation algorithm StarDist^15^. **j**, Violin plot of fraction of nuclei per Visium spot (**g**) for the cortical clusters (**d**) plotted with *P* value annotation. The cluster Cortex_2 was omitted from this analysis because it entails a different region of the cortex (cortical subplate) for which the differential nuclei density score between isocortical layers is not relevant. Test performed was two-sided Mann–Whitney–Wilcoxon test with Bonferroni correction, *P* value annotation legend is the following: ****P ≤ 0.0001. Exact *P* values are the following: Cortex_5 versus Cortex_4, *P* = 1.691 × 10^−36^, *U* = 1,432; Cortex_5 versus Cortex_1, *P* = 2.060 × 10^−54^, *U* = 775; Cortex_5 versus Cortex_3, *P* = 5.274 × 10^−51^, *U* = 787. This example is part of the Squidpy documentation (https://squidpy.readthedocs.io/en/latest/auto_tutorials/tutorial_visium_hne.html).

As previously shown, we can apply spatially variable feature selection to identify genes that show a pronounced spatial pattern. Moran’s *I* spatial correlation statistics identifies *Mobp* and *Nrgn* (Fig. 4a,b) to be spatially variable; both genes show a distinct spatial expression pattern and seem to encompass the localization of several cell clusters (Fig. 4d; ‘Fiber tract’ and ‘Hypothalamus 2’ for *Mobp* and ‘Pyramidal layers’ and ‘Pyramidal layers/Dentate gyrus’ for *Nrgn*). An orthogonal method for the same task, Sepal^42^ ranks *Krt18* as a top spatially variable gene, which shows a distinct expression in a subset of the ‘Lateral ventricle’ cluster (Fig. 4d and Supplementary Fig. 1f,g show a comparison with original implementation). The variety of tools for spatially variable gene identification provided by Squidpy enhances standard cluster-based gene expression signatures by providing insights into spatial distribution of genes. Ligand–receptor interaction analysis can be a useful approach to shortlist candidate genes driving cellular interactions. Squidpy provides a fast re-implementation of the CellphoneDB^43^ method (Supplementary Fig. 1b,d shows runtime comparison against original implementation and Giotto), which additionally leverages the Omnipath database for ligand–receptor annotations^44^ (Supplementary Fig. 1e shows a comparison with CellphoneDB). Applied to the same dataset, it highlighted different ligand–receptor pairs between the ‘Hippocampus’ cluster and the two ‘Pyramidal layer’ clusters. Whether permutation-based tests of ligand–receptor interaction identification are able to pinpoint cellular communication and pathway activity is an open question^45^. However, it is useful to inform such results with a quantitative understanding of cluster co-occurrence. Squidpy’s co-occurrence score is a simple but interpretable approach, which applied to the Visium dataset highlights an expected direct relationship between the previously described clusters (‘Hippocampus’ and the two ‘Pyramidal layer’ clusters; Fig. 4f).

Squidpy’s feature extraction pipeline enables direct comparison and joint analysis of image and omics data. The integrative analysis of gene expression and image data enhances pattern discovery and enables joint interpretation of the information obtained from morphology and molecular data. For instance, on the same mouse brain coronal section data, we compared clusters computed from gene expression profiles with clusters computed from summary statistics (mean, s.d., 0.1, 0.5 and 0.9th quantiles) of high-resolution hematoxylin and eosin (H&E) image channels (Fig. 4g,h). The image-based clusters recapitulate regions of image intensities with similar mean and standard variation, whereas the gene-based clusters are related to broad cell-type definition. We can see that several image-based clusters are highly overlapping with the gene-based clusters, especially in the cluster ‘Hippocampus’ (54% overlap with image feature cluster 10) and the cluster ‘Hypothalamus’ (72% overlap with image feature cluster 8). This shows how members of such clusters share a similar definition both at morphology and molecular level which allows further characterization of the cluster. In contrast, the image-based clusters provide a different view of the data in the cortex (no overlap >33% with any image feature clusters) (Supplementary Fig. 3e). Here, gene clusters identify broad cortical layers whereas the image-based clusters separate different regions of the cortex based on changing local image intensities, indicating changes in cell density, morphology or changes in the staining that are not captured by the gene expression data. For further examination of these image feature clusters, we calculated a nuclei segmentation using StarDist^15^ and extracted the number of nuclei per Visium spot (Fig. 4i). This nuclear count shows that image-based cluster 15 highlights an area in the bottom part of the cortex with low cell density that is not covered fully by the gene cluster ‘Cortex_5’. This example highlights how variation in interpretable image-based features can reveal higher variability within the same annotation and why the integrative tools available in Squidpy enables such analysis. In addition to explaining variation in the image-based clusters, the fraction of nuclei was combined with gene clusters to show that the nuclear density varies between the different cortical clusters (Fig. 4j,). This indicates that gene expression clusters represent a different grouping of the cortex than the one identified by the image-based clustering. Such regions of different nuclear densities and morphology in the brain are of broad interest to neuroscientists^46–48^ and low nuclei density in the outer cortical layer of the isocortex (corresponding to cluster ‘Cortex_5’) has been previously established^48^. Furthermore, Squidpy image-processing tools allow to quickly validate the robustness of such findings, by refining the selection of spots that fully overlap the detected tissue area to remove potential false positives (Supplementary Fig. 6). Therefore, nuclear density and morphological information represent valuable information to disentangle sources of variation in spatial transcriptomics data and allow scientists to generate additional insights for the biological system of interest. Similar tissue hallmarks that can be inferred from image data and may be used to explain gene expression variation, include blood vessels, tissue boundaries and fibrotic areas. Squidpy’s integrative analysis workflows leverage the spatial context and large microscopy images to generate new hypothesis classes in spatial transcriptomics data, thus bridging tissue-level characterizations of samples, which are typical in pathology, with the new high-resolution gene expression characterization yielded by spatial transcriptomics.

## Discussion

In summary, Squidpy enables analysis of spatial molecular data by leveraging two data representations: the spatial graph and the tissue image. Squidpy infrastructure leverages sparse and memory-efficient implementations and its core spatial statistics and image analysis methods are fast and computationally efficient, making them suitable for the increasing size of modern spatial omics data. It interfaces with Scanpy and the Python data science ecosystem, providing a scalable and extendable framework for development of new methods in the field of biological spatial molecular data. Squidpy’s rich documentation in the form of functional application programming interface documentation, examples and tutorial workflows, is easy to navigate and is accessible to both experienced developers and beginner analysts. Furthermore, Squidpy is equipped with an extensive testing suite, implemented in a robust continuous integration pipeline. We foresee in the development roadmap support for GPU-accelerated workflows (specifically using Dask) and a tighter integration with the developing ecosystem of spatial omics methods, with explicit addition of an external module with methods and wrappers provided by contributors and additional tutorials and best practices of the nascent field of spatial omics data analysis. We hope that Squidpy will serve as a bridge between the molecular omics community and the image analysis and computer vision community to develop the next generation of computational methods for spatial omics technologies.

## Methods

### Infrastructure

#### Spatial graph

The spatial graph is a graph of spatial neighbors with cells (or spots in case of Visium) as nodes and neighborhood relations between spots as edges. We use spatial coordinates of spots to identify neighbors among them. Different approaches of defining a neighborhood relation among spots are used for different types of spatial datasets.

Visium spatial datasets have a hexagonal outline for their spots (each spot has up to eight spots situated around it). For this type of spatial dataset the parameter n_rings should be used. It specifies for each spot how many hexagonal rings of spots around it will be considered as neighbors.


sq.gr.spatial_neighbors(adata, coord_type=“grid”, n_neigh=6, n_rings=<int>)


It is also possible to create other types of grid-like graphs, such as squares, by changing the n_neigh argument. For a fixed number of the closest spots for each spot, it leverages the k-nearest neighbors search from Scikit-learn^49^ and n_neigh must be used to set the number of neighbors.


sq.gr.spatial_neighbors(adata, coord_type=“generic”, n_neigh=<int>)


To get all spots within a specified radius (in units of the spatial coordinates) from each spot as neighbors, the parameter radius should be used.


sq.gr.spatial_neighbors(adata, coord_type=“generic”, radius=<float>)


Finally, it is also possible to compute a neighbor graph based on Delaunay triangulation^50^.


sq.gr.spatial_neighbors(adata, coord_type=“generic”, delaunay=True)


The function builds a spatial graph and saves its adjacency and weighted adjacency matrices to adata.obsp[‘spatial_connectivities’] in either Numpy^51^ or Scipy sparse arrays^18^. The weights of the weighted adjacency matrix are distances in the case of coord_type = ‘generic’ and ordinal numbers of hexagonal rings in the case of coord_type = ‘grid’. Together with the connectivities, we also provide a sparse adjacency matrix of distances, saved in adata.obsp[‘spatial_distances’] We also provide spectral and cosine transformation of the adjacency matrix for uses in graph convolutional networks^52^.

#### ImageContainer

ImageContainer is an object for microscopy tissue images associated with spatial molecular datasets. The object is a thin wrapper of an xarray.Dataset^37^ and provides efficient access to in-memory and on-disk images. On-disk files are loaded lazily using dask^19^, meaning content is only read in memory when requested. The object can be saved as a zarr^53^ store. This allows handling of very large files that do not fit in the memory. The images represented by ImageContainer are required to have at least two dimensions, *x* and *y*, with an optional *z* dimension and a variable channels dimension.

ImageContainer is initialized with an in-memory array or a path to an image file on disk. Images are saved with the key layer. If lazy loading is desired, the lazy parameter needs to be specified.


sq.im.ImageContainer(PATH, layer=<str>, lazy=<bool>)


More image layers with the same spatial dimensions *x*, *y* and *z* such as segmentation masks can be added to an existing ImageContainer.


img.add_img(PATH, layer=<str>)


ImageContainer is able to interface with Anndata objects to relate any pixel-level information to the observations stored in Anndata (such as cells and spots). For instance, it is possible to create a lazy generator that yields image crops on-the-fly corresponding to locations of the spots in the image:


spot_generator = img.generate_spot_crops(adata)lambda x: (x for x in spot_generator) # yields crops at spots location


This of course works for both features computed at crop-level but also at segmentation-object level. For instance, it is possible to get centroid coordinates as well as several features of the segmentation object that overlap with the spot capture area.

#### Napari for interactive visualization

Napari is a fast, interactive, multi-dimensional image viewer in Python^22^. In Squidpy, it is possible to visualize the source image together with any Anndata annotation with napari. Such functionality is useful for fast and interactive exploration of analysis results saved in Anndata together with the high-resolution image. If multiple *z* dimensions are available, the individual *z* layers that can be interactively scrolled through. Furthermore, leveraging napari functionalities, it is possible to manually annotate tissue areas and assign underlying spots to annotations saved in the Anndata object. Such ability to relate manually defined tissue areas to observations in Anndata is particularly useful in settings where there is a pathologist annotation available and it needs to be integrated with analysis at gene expression or image level. All the steps described here are performed in Python, therefore available in the same environment where the analysis is performed (it does not require an additional tool).


img = sq.im.ImageContainer(PATH, layer=)img.interactive(adata)


### Graph and spatial patterns analysis

#### Neighborhood enrichment test

The association between label pairs in the connectivity graph is estimated by counting the sum of nodes that belong to classes *i* and *j* (for example cluster annotation) and are proximal to each other, noted *x*_*ij*_. To estimate the deviation of this number versus a random configuration of cluster labels in the same connectivity graph, we scramble the cluster labels while maintaining the connectivities and then recount the number of nodes recovered in each iteration (1,000 times by default). Using these estimates, we calculate expected means (*µ*_*ij*_) and standard deviations (*σ*_*ij*_) for each pair and a *z* score as,

$$Z_{ij} = \left( {x_{ij} - \mu _{ij}} \right)/\sigma _{ij}$$

The *z* score indicates if a cluster pair is over-represented or over-depleted for node–node interactions in the connectivity graph. This approach was described by Schapiro et al.^54^. The analysis and visualization can be performed with the analysis code shown below.


sq.gr.nhood_enrichment(adata, cluster_key=“<cluster_key>”)sq.pl.nhood_enrichment(adata, cluster_key=“<cluster_key>”)


Our implementation leverages just-in-time compilation with Numba^55^ to achieve greater performances in computation time (Supplementary Fig. 1).

#### Ligand–receptor interaction analysis

We provide a re-implementation of the popular CellphoneDB method for ligand–receptor interaction analysis^43^. In short, it is a permutation-based test of ligand–receptor expression across cell-type combinations. Given a list of annotated ligand–receptor pairs, the test computes the mean expression of the two molecules (ligand, receptor) between cell types and builds a null-distribution based on *n* permutations (default 1,000). A P value is computed based on the proportion of the permuted means against the true mean. In CellphoneDB, if a receptor or ligand is composed of several subunits, the minimum expression is considered for the test. In our implementation, we also include the option of taking the mean expression of all molecules in the complex. Our implementation also employs Omnipath^44^ as ligand–receptor interaction database annotation. A larger database that contains the original CellphoneDB database together with five other resources^44^. Finally, our implementation leverages just-in-time compilation with Numba^55^ to achieve greater performances in computation time (Supplementary Fig. 1).

##### Ripley’s spatial statistics

Ripley’s spatial statistics is a family of spatial analysis methods used to describe whether points with discrete annotation in space follow random, dispersed or clustered patterns. Ripley’s statistics can be used to describe the spatial patterning of cell clusters in the area of interest. In Squidpy, we re-implemented three of Ripley’s statistics: *F*, *G* and *L* functions. Ripley’s *L* function is a variance-stabilized transformation of Ripley’s *K* function, defined as

1 $$K\left( t \right) = A\mathop {\sum }\limits_{i = 1}^n \mathop {\sum }\limits_{j = 1}^n I\left( {d_{i,j} < t} \right)$$

Where *I*(*d*_*i*,*j*_ < *t*) is the indicator function that sets whether the operand is 1 or 0 based on the (Euclidean) distance *d*_*i,j*_ evaluated at search radius *t*, *A* is the average density of point in the area of interest. Therefore, the Ripley’s *L* function is defined as:

2 $$L\left( t \right) = \left( {\frac{{K\left( t \right)}}{\pi }} \right)^{1/2}$$

The Ripley’s *F* and *G* functions are defined as:

3 $$P\left( {d_{i,j} \le t} \right)$$

Where *d*_*i,j*_ is the distance of the point to a random points for a spatial Poisson point process for *F* and the distance to any other point of the dataset for *G*. They can be easily computed with:


sq.gr.ripley(adata, cluster_key=“<cluster_key>”, mode=“<F|G|L>”)sq.pl.ripley(adata, cluster_key=“<cluster_key>”, mode=“<F|G|L>”)


##### Cluster co-occurrence ratio

Cluster co-occurrence ratio provides a score on the co-occurrence of clusters of interest across spatial dimensions. It is defined as

4 $$\frac{{p\left( {exp|cluster} \right)}}{{p\left( {exp} \right)}}$$

where cluster is the annotation of interest to be used as conditioning for the co-occurrence of all clusters. It is computed across *n* radius of size *d* across the tissue area. It was inspired by an analysis performed by Tosti et al. to investigate tissue organization in the human pancreas with spatial transcriptomics^56^.


sq.gr.co_occurrence(adata, cluster_key=“<cluster_key>”)sq.pl.co_occurrence(adata, cluster_key=“<cluster_key>”)


##### Spatial autocorrelation statistics

Spatial autocorrelation statistics are widely used in spatial data analysis tools to assess the spatial autocorrelation of continuous features. Given a feature (gene) and spatial location of observations, it evaluates whether the pattern expressed is clustered, dispersed or random^57^. In Squidpy, we implement two spatial autocorrelation statistics: Moran’s *I* and Geary’s *C*.

Moran’s *I* is defined as:

5 $$I = \frac{n}{W}\frac{{\mathop {\sum }\nolimits_{i = 1}^n \mathop {\sum }\nolimits_{j = 1}^n w_{i,j}z_iz_j}}{{\mathop {\sum }\nolimits_{i = 1}^n z_i^2}}$$

and Geary’s *C* is defined as:

6 $$C = \frac{{\left( {n - 1} \right)\mathop {\sum }\nolimits_{i,j} w_{i,j}\left( {x_i - x_j} \right)^2}}{{2W\mathop {\sum }\nolimits_i \left( {x_i - \bar x} \right)^2}}$$

where *z*_*i*_ is the deviation of the feature from the mean $\left( {x_i - \bar X} \right)$, *w*_*i,j*_ is the spatial weight between observations, *n* is the number of spatial units and *W* is the sum of all *w*_*i,j*_. Test statistics and *P* values (computed from a permutation-based test or via analytic formulation, similar to libpysal^58^ and further FDR-corrected) are stored in adata.uns[‘moranI’] or adata.uns[‘gearyC’].


sq.gr.spatial_autocorr(adata, cluster_key=“<cluster_key>”,mode=“<moran|geary>”)


##### Sepal

Sepal is a recently developed method for spatially variable genes identification^42^. It simulates a diffusion process and evaluates the time it takes to reach a uniform state (convergence). It is a formulation of Fick’s second law to a regular graph (grid). It is defined as:

7 $$u\left( {x,y,t + dt} \right) = u\left( {x,y,t} \right) + D{\Delta}u\left( {x,y,t} \right)dt$$

Where *u*(*x,y,t*) is the concentration (for example gene expression on a node in *x*,*y* coordinates), *D* is the diffusion coefficient, *t* is the update time and *∆*(*u*(*x,y,t*)*dt*) is the laplacian on the graph (see elsewhere^42^ for an extended formulation). Convergence is reached if the change in entropy is below a given threshold:

8 $$H\left( {u\left( t \right)} \right) - H\left( {u\left( {t - 1} \right)} \right) < {\it{\epsilon }}$$

The time *t* the gene takes to reach consensus is then used a ‘Sepal score’ and indicates the degree of spatial variability of the gene. It can be computed with:


sq.gr.sepal(adata)


Our re-implementation in Numba achieves greater computational efficiency (Supplementary Fig. 1).

##### Centrality scores

Centrality scores provide a numerical analysis on node patterns in the graph, which helps to better understand complex dependencies in large graphs. A centrality is a function *C* which assigns every vertex *v* in the graph a numeric value *C*(*v*) ∈ R. It therefore gives a ranking of the single components (cells) in the graph, which simplifies to identify key individuals. Group centrality measures have been introduced by Everett and Borgatti^59^. They provide a framework to assess clusters of cells in the graph (a specific cell type more central or more connected in the graph than others). Let *G* = (*V,E*) be a graph with nodes *V* and edges *E*. Additionally, let *S* be a group of nodes allocated to the same cluster *c*_*S*_. Then *N*(*S*) defines the neighborhood of all nodes in *S*. The following four (group) centrality measures are implemented. Group degree centrality is defined by the fraction of noncluster members that are connected to cluster members, so

$$C_{deg}\left( S \right) = \frac{{\left| {N\left( S \right) - S} \right|}}{{\left| V \right| - \left| S \right|}} \in \left[ {0,1} \right]$$

Larger values indicate a more central cluster. Group degree centrality can help to identify essential clusters or cell types in the graph. Group closeness centrality measures how close the cluster is to other nodes in the graph and is calculated by the number of nongroup members divided by the sum of all distances from the cluster to all vertices outside the cluster, so

$$C_{clos}\left( S \right) = \frac{{\left| {V - S} \right|}}{{\mathop {\sum }\nolimits_{v \in V_S} d_{S,v}}} \in \left[ {0,1} \right]$$

where *d*_*S*,*v*_ = min_*u*∈*S*_ *d*_*u*,*v*_ is the minimal distance of the group *S* from *v*. Hence, larger values indicate a greater centrality. Group betweenness centrality measures the proportion of shortest paths connecting pairs of nongroup members that pass through the group. Let *S* be a subset of a graph with vertex set *V*_*S*_. Let *g*_*u*,*v*_ be the number of shortest paths connecting *u* to *v* and *g*_*u*,*v*_(*S*) be the number of shortest paths connecting *u* to *v* passing through *S*. The group betweenness centrality is then given by

$$C_{betw}\left( S \right) = \mathop {\sum }\limits_{u < v} \frac{{g_{u,v}\left( S \right)}}{{g_{u,v}}}{{{\mathrm{for}}}}\,u,v \notin S.$$

The properties of this centrality score are fundamentally different from degree and closeness centrality scores, hence results often differ. The last measure described is the average clustering coefficient. It describes how well nodes in a graph tend to cluster together. Let *n* be the number of nodes in *S*. Then the average clustering coefficient is given by

$$C_{cluster}\left( S \right) = \frac{1}{n}\mathop {\sum }\limits_{v \in S} \frac{{2T\left( v \right)}}{{deg\left( v \right)\left( {deg\left( v \right) - 1} \right)}}$$

with *T*(*v*) being the number of triangles through node *v* and *deg*(*v*) the degree of node *v*. The described centrality scores have been implemented using the NetworkX library in Python^50^.


sq.gr.centrality_scores(adata, cluster_key=“<cluster_key>”)sq.pl.centrality_scores(adata, cluster_key=“<cluster_key>”, selected_score=“<selected_score>”)


Interaction matrix represents the total number of edges that are shared between nodes with specific attributes (such as clusters or cell types).


sq.gr.interaction_matrix(adata, cluster_key=“<cluster_key>”, normalized=True)sq.pl.interaction_matrix(adata, cluster_key=“<cluster_key>”)


Python implementations rely on the NetworkX library^50^.

### Image analysis and segmentation

#### Image processing

Before extracting features from microscopy images, the images can be preprocessed. Squidpy implements functions for commonly used preprocessing functions like conversion to grayscale or smoothing using a Gaussian kernel. In addition, custom processing functions can be used by passing a function to the method argument.


sq.im.process(img, method=“gray”)img.show()


Implementations are based on the Scikit-image package^21^ and allow lazy processing of very large images through tiling the image into smaller crops and processing these by using Dask. When using tiling, image crops are slightly overlapping, to reduce border effects.

#### Image segmentation

Nuclei segmentation is an important step when analyzing microscopy images. It allows the quantitative analysis of the number of nuclei, their areas and morphological features. There are a wide range of approaches for nuclei segmentation, from established techniques such as thresholding to modern deep-learning-based approaches.

A difficulty for nuclei segmentation is to distinguish between partially overlapping nuclei. Watershed is a classic algorithm used to separate overlapping objects by treating pixel values as local topology. For this, starting from points of lowest intensity, the image is flooded until basins from different starting points meet at the watershed ridge lines.


sq.im.segment(img, method=“watershed”)img.show()


Implementations in Squidpy are based on the original Scikit-image Python implementation^21^.

#### Custom approaches with deep-learning

Depending on the quality of the data, simple segmentation approaches like watershed might not be appropriate. Nowadays, many complex segmentation algorithms are provided as pretrained deep-learning models, such as Stardist^15^, Splinedist^60^ and Cellpose^16^. These models can be easily used within the segmentation function. We provide extensive tutorials https://squidpy.readthedocs.io/en/latest/tutorials.html#external-tutorials, where we show how Stardist^15^ and Cellpose^16^ can be easily interfaced with Squidpy to perform segmentation on both H&E and fluorescence images.


sq.im.segment(img, method=<pre-trained model>)img.show()


#### Image features

Tissue organization in microscopic images can be analyzed with different image features. This filters relevant information from the (high-dimensional) images, allowing for easy interpretation and comparison with other features obtained at the same spatial location. Image features are calculated from the tissue image at each location (*x*,*y*) where there is transcriptomics information available, resulting in an obs × features matrix similar to the obs × gene matrix. This image feature matrix can then be used in any single-cell analysis workflow, just like the gene matrix.

The scale and size of the image used to calculate features can be adjusted using the scale and spot_scale parameters. Feature extraction can be parallelized by providing n_jobs (see Supplementary Fig. 1). The calculated feature matrix is stored in adata[key].


sq.im.calculate_image_features(adata, img, features=<list>, spot_scale=<float>, scale=<float>, key_added=<str>)


Summary features calculate the mean, the s.d. or specific quantiles for a color channel. Similarly, histogram features scan the histogram of a color channel to calculate quantiles according a defined number of bins.


sq.im.calculate_image_features(adata, img, features=“summary”) sq.im.calculate_image_features(adata, img, features=“histogram”)


Textural features calculate statistics over a histogram that describes the signatures of textures. To grasp the concept of texture intuitively, the inextricable relationship between texture and tone is considered^61^; if a small-area patch of an image has little variation in its gray tone the dominant property of that area is tone. If the patch has a wide variation of gray-tone features, the dominant property of the area is texture. An image has a simple texture if it consists of recurring textural features. For a gray-level image I or for example a fluorescence color channel, a co-occurrence matrix C is computed. C is a histogram over pairs of pixels (*i*,*j*) with specific values (*p*,*q*) ∈ [0,1*,…*,255] (https://squidpy.readthedocs.io/en/latest/tutorials.html#external-tutorials) and a fixed pixel offset:

$$C_{p,q} = \mathop {\sum }\limits_i \delta _{I\left( i \right),p}\delta _{I\left( j \right),q}$$

with Kronecker delta *δ*. The offset is a fixed pixel distance from *i* to *j* under a fixed direction angle. Based on the co-occurrence matrix different meaningful statistics (texture properties) can be calculated that summarize textural pattern characteristics of the image:

- $$\mathop {\sum }\limits_{p,q} C_{p,q}\left( {p - q} \right)^2$$ contrast
- $$\mathop {\sum }\limits_{p,q} C_{p,q}\left| {p - q} \right|$$ dissimilarity
- $$\mathop {\sum }\limits_{p,q} \frac{{C_{p,q}}}{{1 + \left( {p - q} \right)^2}}$$ homogeneity
- $\mathop {\sum }\limits_{p,q} C_{p,q}^2$ angular second moment
- $$\mathop {\sum }\limits_{p,q} C_{p,q}\frac{{\left( {p - \mu _p} \right)\left( {q - \mu _q} \right)}}{{\sqrt {\sigma _p^2\sigma _q^2} }}$$ correlation

sq.im.calculate_image_features(adata, img, features=“texture”)


All the above implementations rely on the Scikit-image Python package^21^.

#### Segmentation features

Similar to image features that are extracted from raw tissue images, segmentation features can be extracted from a segmentation object. These features allow to get statistics over the number, area and morphology of the nuclei in one image. To compute these features, the ImageContainer img needs to contain a segmented image at layer <segmented_img>


sq.im.calculate_image_features(adata, img,features=“segmentation”, features_kwargs={“label_layer”:<segmented_img>})


#### Custom features based on deep-learning models

Squidpy feature calculation function can also be used with custom user-defined features extraction functions. This enables the use of for example, pretrained deep-learning models as feature extractors. We provide tutorials https://squidpy.readthedocs.io/en/latest/tutorials.html#external-tutorials on how to interface popular deep-learning frameworks such as Tensorflow^62^ with ImageContainer, thus enabling users to perform an end-to-end deep-learning pipeline from Squidpy.


sq.im.calculate_image_features(adata, img, features=“custom”, features_kwargs={“func”:<pre-trained keras model>})


### Reporting Summary

Further information on research design is available in the Nature Research Reporting Summary linked to this article.

## Online content

Any methods, additional references, Nature Research reporting summaries, source data, extended data, supplementary information, acknowledgements, peer review information; details of author contributions and competing interests; and statements of data and code availability are available at 10.1038/s41592-021-01358-2.

## Supplementary information

Supplementary Information Supplementary Figs. 1–6 Supplementary methods.

Reporting Summary

Peer Review Information

Supplementary Table 1 and 2 File with two tables.

## Data Availability

The preprocessed datasets have been deposited at 10.6084/m9.figshare.c.5273297.v1 and they are all conveniently accessible in Python via the squidpy.dataset module. The datasets used in this article are the following: Imaging Mass Cytometry^36^, seqFISH^33^, 4i^31^, MERFISH^34^, SlideseqV2 (ref. ^35^), Mibi-tof^41^ and several Visium^24^ datasets available from https://support.10xgenomics.com/spatial-gene-expression/datasets. Information on preprocessing of such datasets can be found in Online Methods and code to reproduce it is at https://github.com/theislab/squidpy_reproducibility.

## Code availability

Squidpy is a pip installable Python package and available at the following GitHub repository: https://github.com/theislab/squidpy, with documentation at: https://squidpy.readthedocs.io/en/latest/. All the code to reproduce the result of the analysis can be found at the following GitHub repository: https://github.com/theislab/squidpy_reproducibility.
