# Supplementary material for: Squidpy: a scalable framework for spatial omics analysis
Source: Nat Methods. 2022 Jan 31;19(2):171–8. doi: 10.1038/s41592-021-01358-2 (PMC8828470; doi:10.1038/s41592-021-01358-2)
Supplement: Supplementary file 1 — Supplementary Figs. 1–6 Supplementary methods. [file 41592_2021_1358_MOESM1_ESM.pdf]

nature portfolio
https://doi.org/10.1038/s41592-021-01358-2

Supplementary information

Squidpy: a scalable framework for spatial omics analysis

In the format provided by the authors and unedited

Squidpy - Supplementary

Giovanni Palla*[1,2], Hannah Spitzer*[1], Michal Klein[1], David Fischer[1,2], Anna Christina Schaar[1,2], Louis Benedikt Kuemmerle[1,4], Sergei Rybakov[1,3], Ignacio L. Ibarra[1], Olle Holmberg[1], Isaac Virshup[5], Mohammad Lotfollahi[1,2], Sabrina Richter[1,2], Fabian J. Theis[1,2,3+]

1 Institute of Computational Biology, Helmholtz Center Munich, Germany.
2 TUM School of Life Sciences Weihenstephan, Technical University of Munich, Germany.
3 Department of Mathematics, Technical University of Munich, Germany.
4 Institute for Tissue Engineering and Regenerative Medicine (iTERM), Helmholtz Center Munich, Germany
5 Department of Anatomy and Physiology, University of Melbourne, Australia.

*equal contribution
[+]Correspondence: fabian.theis@helmholtz-muenchen.de

Supplementary Information

Dataset pre-processing

All the datasets analyzed in the manuscript can be easily accessed with the Squidpy API. The following code will trigger the download of pre-processed datasets from the figshare repository. For example, to download the seqFISH dataset:

```
adata = sq.datasets.seqfish()
```

Otherwise, to download both Anndata and large tissue image of the HE mouse brain dataset from 10X genomics:

```
adata = sq.datasets.visium_hne_adata()
img = sq.datasets.visium_hne_image()
```

The datasets were obtained from original publications/resources and a very minor processing/filtering has been performed before being included in Squidpy. Specifically, for each dataset, we performed the following processing:

- 10X Genomics Visium data: data was downloaded from https://www.10xgenomics.com/resources/ datasets. Normalization and filtering was performed with standard Scanpy analysis tools[1]. Clustering was performed with the leiden algorithm[2] and cell-type was annotation by qualitatively assessing cluster similarity with the Allen Brain Atlas portal https://portal.brain-map.org/.
- seqFISH data[3]: no additional processing was performed.
- Imaging Mass Cytometry[4]: no additional processing was performed.
- MERFISH[5]: no additional processing was performed.
- MIBI-TOF[6]: 3 tissue samples were selected from the interactive image viewer at https:// mibi-share.ionpath.com/tracker/login/. Features across these 3 samples were standardized with Scanpy and batch-corrected with Scanorama[7]. Segmentation masks were downloaded from Zenodo and saved in the same AnnData object: https://zenodo.org/record/3951613/export/ hx#.YL4q9jYzY7w
- SlideseqV2[8]: original raw data were downloaded from https://singlecell.broadinstitute. org/single_cell. Standard Scanpy processing pipeline was performed (filtering,

normalization and highly variable genes selection). Cell type annotation and deconvolution results were kindly provided by the original authors.

Squidpy neighborhood analysis application to MERFISH and seqFISH.

In Figure 2b we applied Squidpy neighborhood enrichment analysis tool (1000 permutations, see online methods) between cell clusters in spatial coordinates (spatial graph built with Delaunay triangulation method). Qualitative differences between analyses could arise due to the number of permutations employed (ours: 1000 permutations, original authors: 500 permutations) and construction of spatial graphs (ours: Delaunay triangulation, original authors: custom method leveraging segmentation masks). The neighborhood enrichment score retrieves interactions for annotations both clustered ("lateral plate mesoderm" with "Allantois" and "Intermediate mesoderm" but also dispersed ("Haematoendothelial progenitors" and "Endothelium") across the tissue area.

In Figure 2e we applied Squidpy neighborhood enrichment analysis tool (1k permutations, see online methods) of the MERFISH dataset in 3D (spatial graph built with the kNN method).

Supplementary Figures

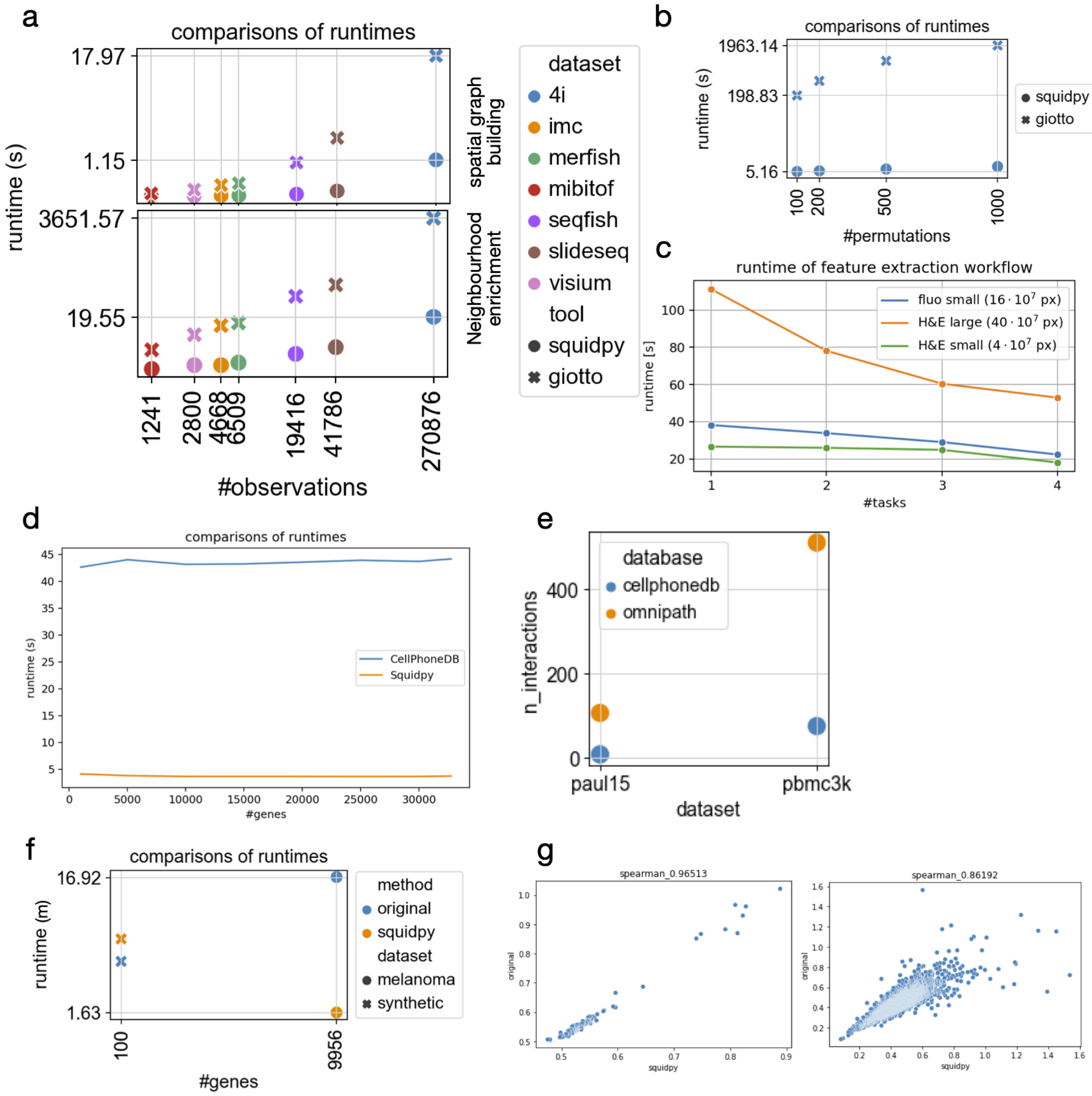

Supplementary Figure 1. Benchmarking resources for Squidpy analysis modules.

Benchmarks (a), (b), (f) were run on a 2,4 GHz Intel Core i5 processor with 8 cores and 16 GB RAM. Benchmarks (c) and (d) were run on a Centos 8 server cluster with 32 cores and 128 GB of memory. Unless explicitly mentioned, functions were run without parallelization.

(a) Execution times for spatial graph building and neighborhood enrichment analysis, comparing seven spatial datasets at increasing number of observations. Squidpy outperforms similar functions provided by the Giotto toolkit[9]. In particular, reporting a minimum of 12-fold and a maximum of 15-fold speedup for the graph construction step, and a minimum of 8-fold and a maximum of 187-fold speedup for the neighborhood enrichment step. Reported are mean values for 10 runs, except for the 4i, SlideseqV2 and Seqfish neighbor enrichment test that was run only once in Giotto due to computational time demands. Axes are in log10 scale.

(b) Execution time for permutation test of ligand-receptor interaction analysis for one dataset (2800 observations, 15 clusters, 599 ligand-receptor pairs) at increasing number of permutations, computed using 1 thread (although Squidpy could parallelize easily, see (d)). Squidpy shows almost constant execution time (<7 seconds for 1000 permutations) compared to exponential increase for Giotto. It

should be noted that the ligand-receptor pairs were the ones provided by Giotto ("mouse_ligand_receptors.txt" file), and 600 pairs were found in the dataset. Squidpy interface with Omnipath is able to retrieve 9014 interactions for the same dataset (data not shown), which corresponds to a 15X increase in available annotations. Axis is in log10 scale.
(c) Execution time for typical feature extraction workflow on different datasets. The feature extraction workflow consisted of segmenting the image using watershed with a fixed threshold, and extracting summary and segmentation features with default parameters. The segmentation was done using image tiles of size 2000. Using more cores (tasks) linearly decreases computation time for the feature extraction workflow, enabling processing of very large images (>400M pixels).
(d) Execution time for Squidpy's implementation of the CellphoneDB permutation-based test, at an increasing number of genes for the development of human forebrain dataset[10].
(e) Squidpy implementation of the CellphoneDB permutation-based tests uses the full Omnipath database for ligand receptor annotations. For two datasets (paul15[11] mouse and pbmc3k[12] human), Omnipath in Squidpy can recover a higher number of interactions.
(f) Execution time for permutation Sepal score (original implementation vs Squidpy) for two datasets (synthetic and melanoma) from the original Sepal article (for the first dataset, computation was performed on 1 core for both methods, for the second dataset, computation was performed on 8 cores for both methods)[13].
(g) Correlations of Sepal score between original implementation and Squidpy: 0.96 and 0.86 for the synthetic and melanoma datasets respectively.

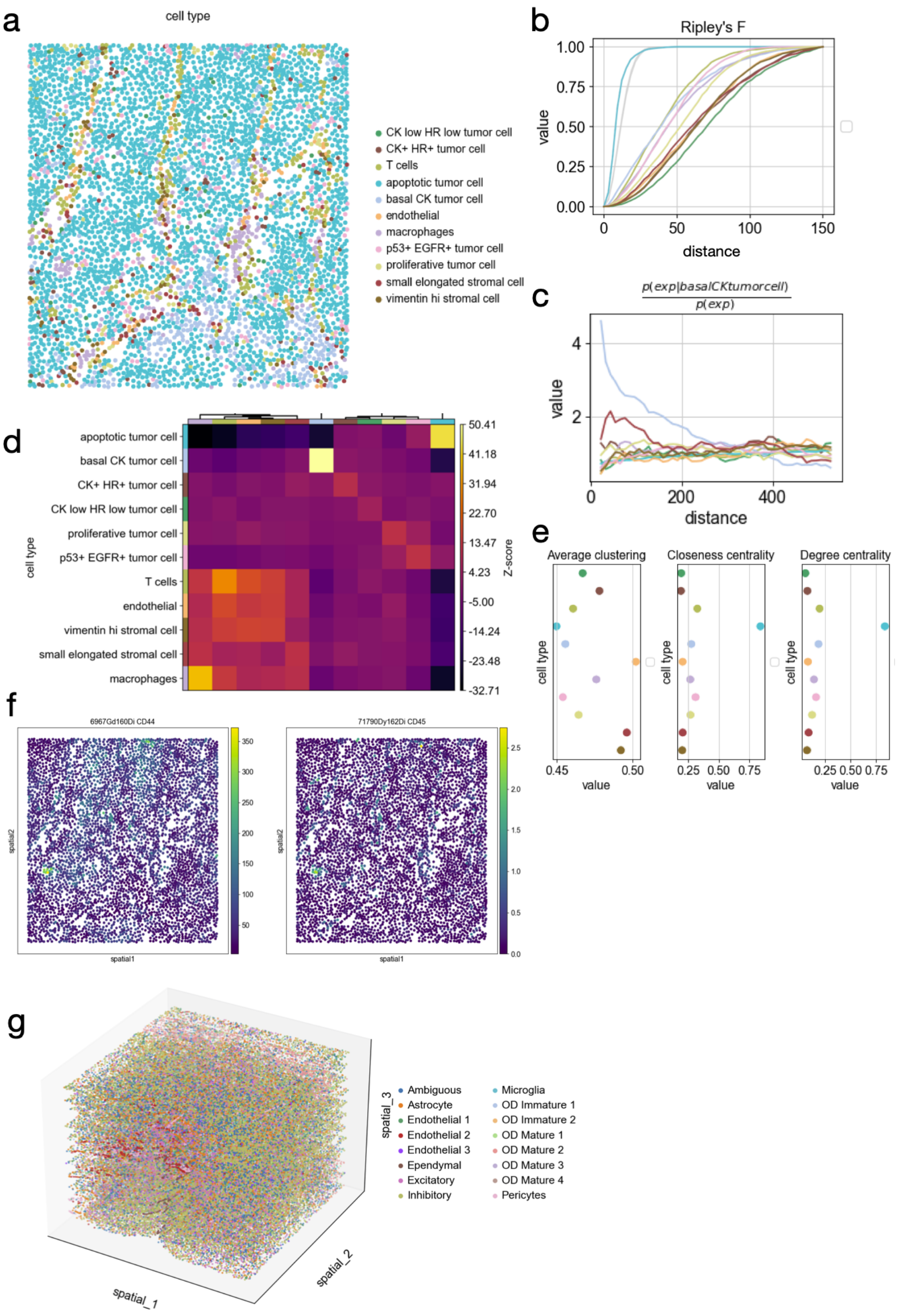

Supplementary Figure 2. Example analysis of Imaging Mass Cytometry data from breast cancer biopsies.

(a) Spatial visualization of cell types in the Imaging Mass Cytometry dataset, as defined by the original authors[4].
(b) Ripley's F statistics computed at increasing distances threshold across the tissue. The gray-shaded line represents the Spatial Poisson point process baseline. All the clusters are below the baseline, thus showing a dispersed pattern across the area. "Apoptotic tumor cell" is the only cluster that shows a pattern similar to the random baseline.
(c) Co-occurrence analysis of cell types at increasing distance thresholds across the tissue. Visualized is the probability conditioned on the presence of the "basal CK tumor cell". Interestingly, we can observe a slight co-enrichment with the "small elongated stromal cell" cluster.
(d) Neighborhood enrichment analysis between cell type clusters in the spatial graph. We can observe how the immune cell subsets and stromal cells seem to form a closer neighborhood as opposed to the tumor cells.

(e) Network centralities for cell types (nodes of the spatial graph). The "apoptotic tumor cell" cluster shows high closeness and degree centrality, and it is indeed the most abundant and spread class label in the graph.
(f) Visualization of two markers for immune cell populations, visualized in spatial coordinates.
(g) Visualization of the full MERFISH dataset as described in Figure 2.

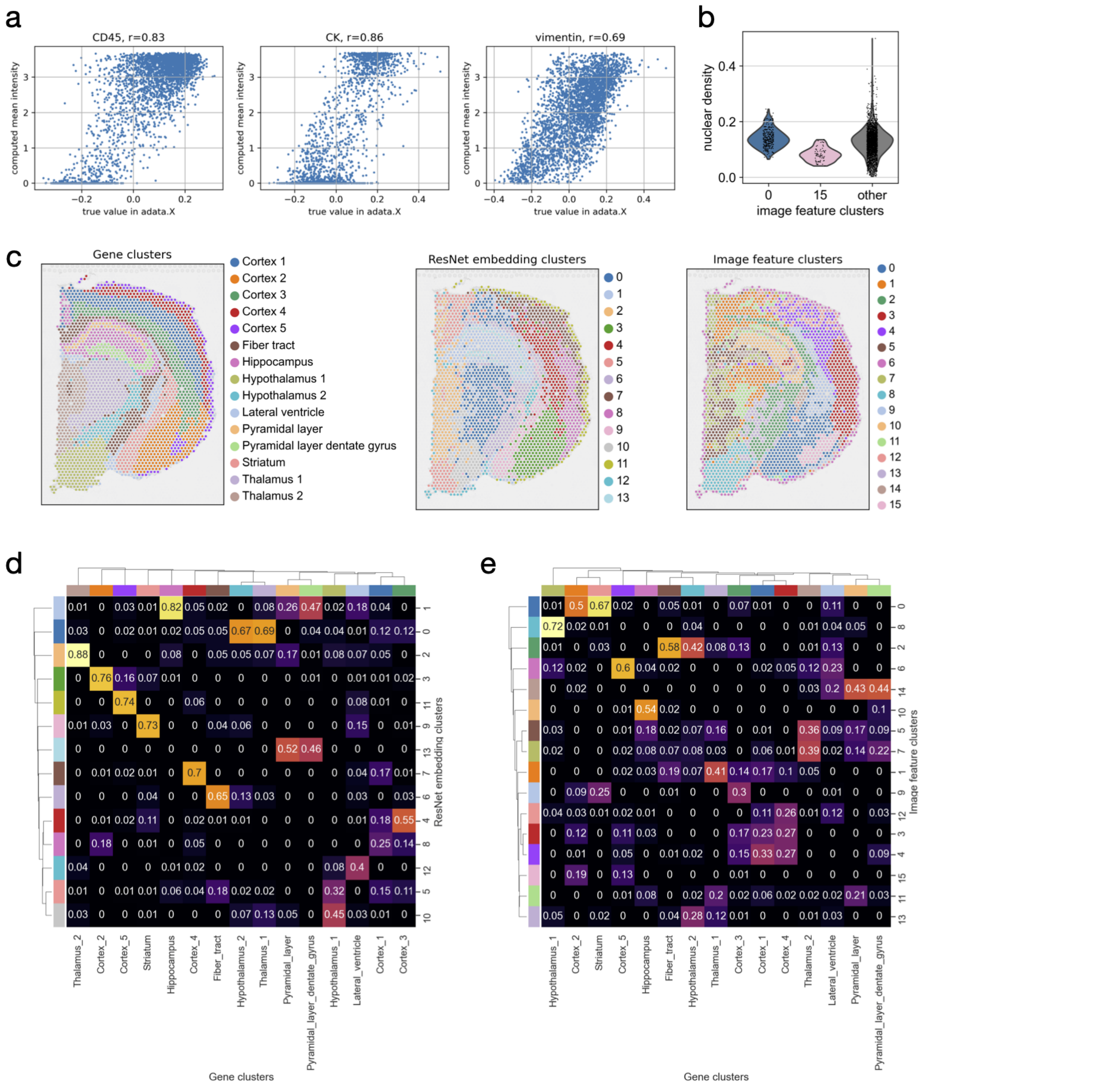

Supplementary Figure 3. Additional image analysis examples

(a) Quantitative comparison of results from the Mibi-tof dataset[6] (Fig. 3f). Shown are scatterplots relating computed mean intensities (Fig. 3f center right and right) to true intensity values in the original publication. For all markers, there is a high correlation between computed and true values (Pearson correlation >= 0.69). The remaining differences are most likely due to more preprocessing and per-slide normalisation applied in the original manuscript[6].
(b) Violinplot of nuclear density in image feature clusters calculated using summary features on an H&E mouse brain section (Fig. 4h). Cluster 15 contains less nuclei than the surrounding cluster 0.
(c) Qualitative comparison of gene-space clustering (left) with clustering of ResNet features (center) and clustering of summary features (right, see Fig. 4h) using a mouse brain Visium dataset with an H&E microscopy image. ResNet features were calculated by training a pre-trained ResNet model to predict the gene-expression cluster assignment (shown on the left) and taking the feature vector of the last fully connected layer as data representation.
(d) Confusion matrix showing the proportion of assigned labels in gene clusters and resnet embedding clusters from (c). Rows correspond to clusters in gene expression space (c) left),

columns correspond to resnet embedding clusters (c) center). The heatmap shows the proportion of overlapping observations in each cluster annotation. For instance, for "Thalamus_2" cluster, 88% of observations are annotated as cluster 2 in the resnet embedding visualization. We can see that for some cluster labels the prediction was strong, whereas for others the resnet model was unable to discriminate the labels. For instance, some regions of the cortex and hypothalamus seemed to not have been accurately classified. This showcases how the image container object can be used to relate morphology information from the source image to any annotation in the Anndata object.
(e) Confusion matrix showing the proportion of assigned labels in gene clusters and image summary feature clusters from (c). Rows correspond to clusters in gene expression space (c) left), columns correspond to image summary feature clusters (c) center). The heatmap shows the proportion of overlapping observations in each cluster annotation. Several of the gene clusters are recognizable using simple image features. E.g.,"Hypothalamus_1" is overlapping to 72% with cluster 8, "Hippocampus" is overlapping to 54% with cluster 10, and "Pyramidial_layer" and "Pyramidial_layer_dentate_gyrus" are covered to 43%/44% by cluster 14. In other regions, especially the cortex (clusters "Cortex_1", "Cortex_3", "Cortex_4"), the image clusters do not overlap well (no cluster overlap > 33%), showing that in these regions simple image features and gene expression values show different patterns.

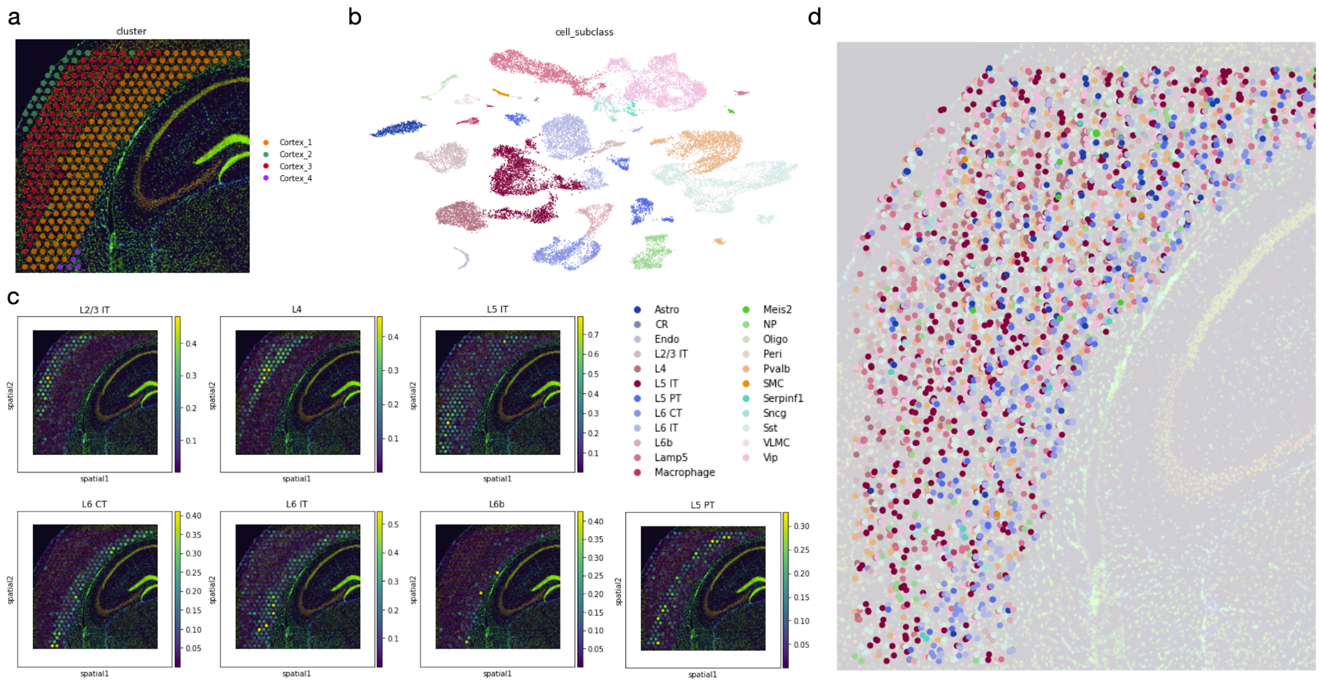

Supplementary Figure 4. Interfacing Squidpy to Tangram for segmentation-aware cell-type deconvolution.

Squidpy’s Image Container can be used to acquire nuclei's segmentation masks and derive cell densities. Priors on nuclei densities can be leveraged by deconvolution methods such as Tangram[14]. Such information is not only helpful for the cell-type mapping task, but also for further downstream visualization, where each segmentation object can be labelled by the inferred cell state (see d).
(a) Subset of Visium spatial transcriptomics dataset showing a mouse brain coronal section.
(b) Matched scRNA-seq data from the mouse cortex from Tasic et al[15].
(c) Tangram results as averaged by cell type. Tangram successfully assigns cortical-layer specific cell-types to spatially distinct layers of the cortex
(d) Tangram maps of single cells. The cell type of the segmentation objects were assigned by Tangram, employing the seamless integration provided by Squidpy between the segmentation objects coordinates and the coordinates original spot observations in Anndata. In the figure, each point corresponds to a segmentation object colored by the cell type assigned by Tangram.
This example is part of the Squidpy documentation (see https://squidpy.readthedocs.io/en/latest/external_tutorials/tutorial_tangram.html).

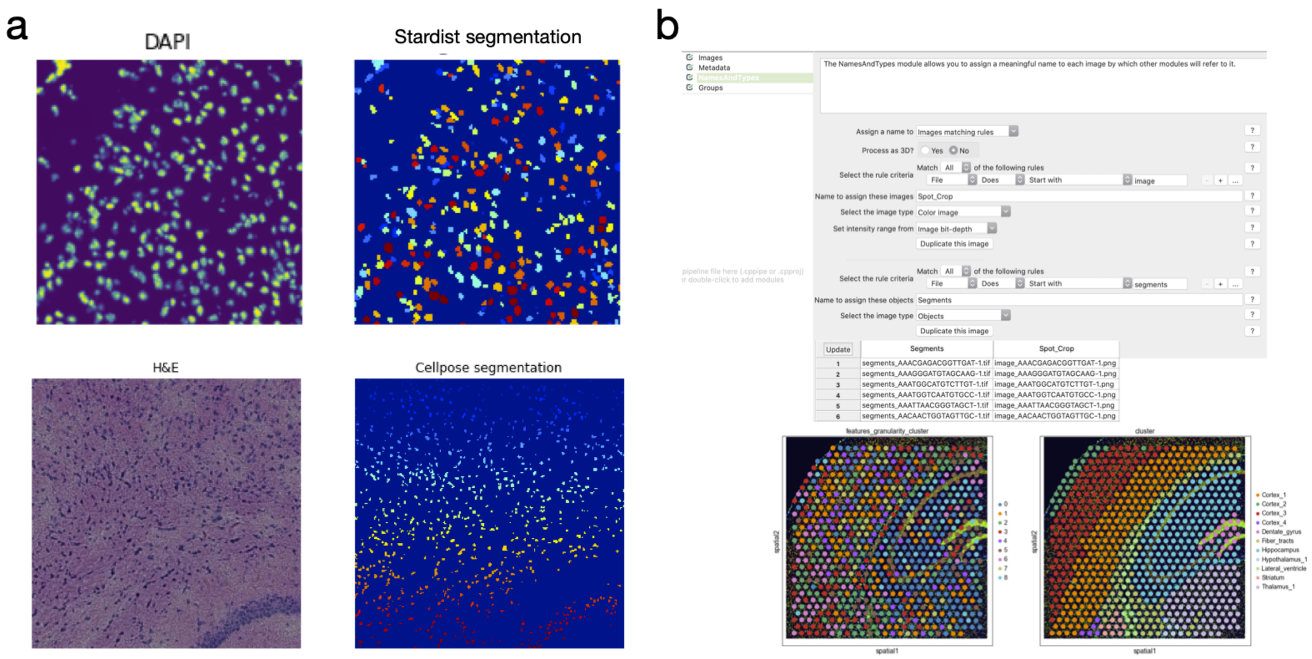

Supplementary Figure 5. Interfacing Squidpy to DL-based segmentation methods and CellProfiler.

(a) Segmentation results for DAPI staining with StarDist (top) and for H&E staining with Cellpose (bottom). Squidpy's Image Container is flexible to be interfaced with modern DL-based models. Both of these examples are part of the Squidpy documentation (see link in code and data availability sections).

(b) Example of CellProfiler pipeline interfaced with Squidpy: here, the CellProfiler pipeline performs segmentation and computes segmentation-masks features such as granularity, which is then used downstream by Squidpy to obtain cluster annotation (bottom-left clusters, compared to gene-clusters in bottom-right of the figure). This example is part of the Squidpy documentation (see https://squidpy.readthedocs.io/en/stable/external_tutorials/tutorial_cellpose_segmentation.htmll, https://squidpy.readthedocs.io/en/stable/external_tutorials/tutorial_stardist.html, and https://squidpy.readthedocs.io/en/stable/external_tutorials/tutorial_cellprofiler.html).

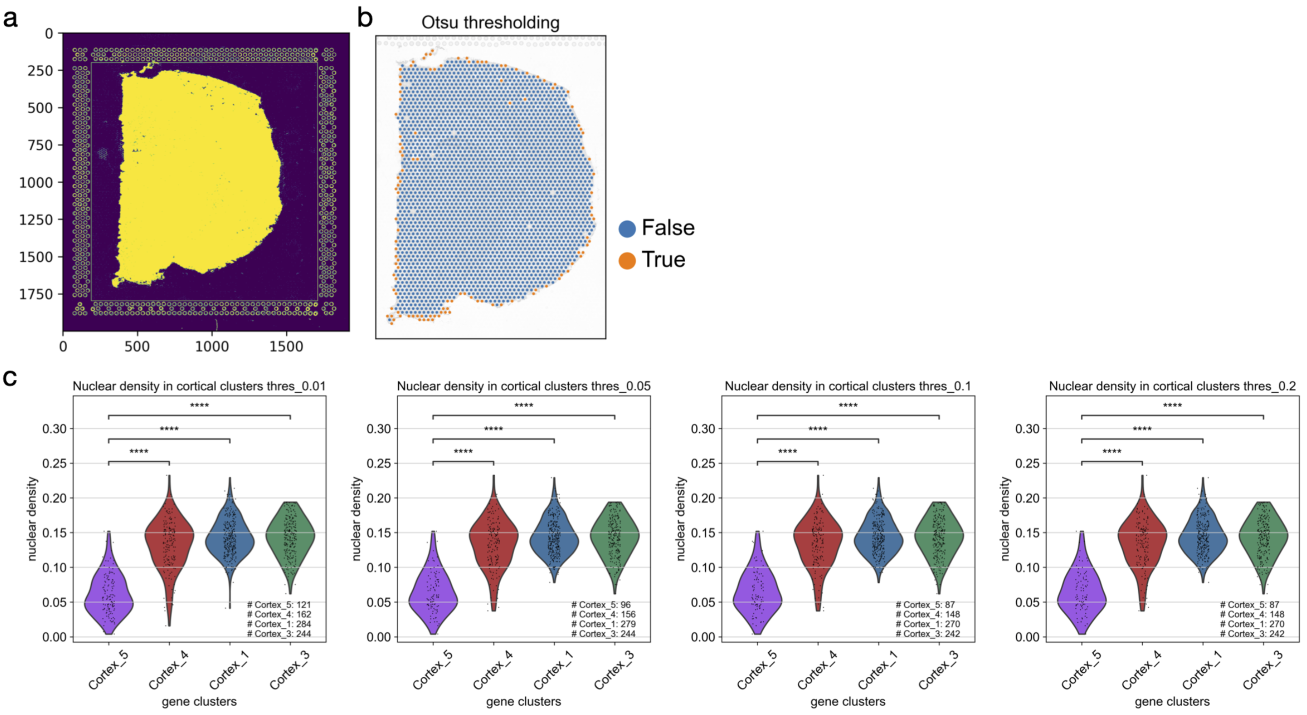

Supplementary Figure 6. Thresholding the tissue image to filter spots that do not fully overlap with detected tissue does not impact differential nuclei density between cortical layers.

(a) Visualization of Otsu's thresholding of Visium slide (detected tissue and fiducial spots). The resulting image is a binarized version based on the threshold selected by Otsu's method. Yellow pixels are 1 whereas blue pixels are 0.
(b) Spots filtered based on 0.05 quantile on Otsu's thresholding density. Orange spots are spots that are filtered by the threshold, since they do not retain a mean intensity value that is above the selected quantile (0.05). Blue spots are instead kept for downstream analysis, as they retain high overlap with the detected tissue. This shows that most of the removed spots are the ones at the boundary of the tissue, only partially overlapping with the detected tissue.
(c) Same analysis from Figure 4j, now performed at increasing quantile values for the Otsu's thresholding from (b). From left to right, nuclei density estimation is performed only including spots that are above the selected quantile value: 0.01, 0.05, 0.1, 0.2. Interestingly, despite the increased number of spots filtered out at increasing quantile values, the high-confidence spots of cluster "Cortex_5" still show a significant decrease in nuclei density as compared to the other cortical layers. In the figure annotation, absolute number of spots for each cluster is reported, notice that the absolute number decreases at increased quantile's thresholding value. The result without Otsu's thresholding is reported in the updated Figure 4.j.
Test performed is Mann-Whitney-Wilcoxon test two-sided with Bonferroni correction, p-value annotation legend is the following:
ns: 5.00e-02 < p <= 1.00e+00
*: 1.00e-02 < p <= 5.00e-02
**: 1.00e-03 < p <= 1.00e-02
***: 1.00e-04 < p <= 1.00e-03
****: p <= 1.00e-04
For 0.01 exact p values are the following:
Cortex_5 v.s. Cortex_4: P_val=5.931e-35 U_stat=1.340e+03
Cortex_5 v.s. Cortex_1: P_val=7.776e-52 U_stat=7.700e+02
Cortex_5 v.s. Cortex_3: P_val=1.248e-48 U_stat=7.840e+02

For 0.05 exact p values are the following:
Cortex_5 v.s. Cortex_4: P_val=2.902e-30 U_stat=1.010e+03
Cortex_5 v.s. Cortex_1: P_val=1.471e-43 U_stat=6.410e+02
Cortex_5 v.s. Cortex_3: P_val=8.409e-41 U_stat=7.325e+02
For 0.1 exact p values are the following:
Cortex_5 v.s. Cortex_4: P_val=1.737e-27 U_stat=9.210e+02
Cortex_5 v.s. Cortex_1: P_val=4.908e-40 U_stat=5.890e+02
Cortex_5 v.s. Cortex_3: P_val=1.706e-37 U_stat=7.245e+02
For 0.2 exact p values are the following:
Cortex_5 v.s. Cortex_4: P_val=1.737e-27 U_stat=9.210e+02
Cortex_5 v.s. Cortex_1: P_val=4.908e-40 U_stat=5.890e+02
Cortex_5 v.s. Cortex_3: P_val=1.706e-37 U_stat=7.245e+02

Supplementary Table 1. Comparison of Squidpy features to existing tools for spatial molecular data analysis

Rows correspond to a set of analysis features that are specific for working with spatial molecular data. It is subdivided in Infrastructure, Spatial Analysis, Image Analysis, Integration, Visualization and Others. The columns contain software tools that are tailored for spatial data analysis: Squidpy, stLearn[16], Giotto[9], Seurat[17], SpatialExperiment[18], STutility[19], TissUUmaps[20]. Entries have been labelled according to whether the software tool is able to provide a specific functionality, whether it's partially available or whether it's missing. The row “Framework” specifies which programming languages are necessary to use all of the functionalities of the package. Finally, for SpatialExperiment, since it is an object to store spatial transcriptomics data, the analysis features do not apply.

Supplementary Table 2. Comparison of available methods between Giotto and Squidpy.

Comprehensive comparison between methods provided by the Giotto package and Squidpy, grouped by analysis tasks. Implementation details between functions may differ but are deemed to perform the same analysis. The two frameworks are largely similar with respect to general tasks such as visualization. Giotto has a richer set of functions for the spatial graph but does not provide any methods to process and analyze the large tissue image, a modality often present in spatial omics data.

References

1. Wolf, F. A., Angerer, P. & Theis, F. J. SCANPY: large-scale single-cell gene expression data analysis. *Genome Biol.* **19**, 15 (2018).
2. Traag, V. A., Waltman, L. & van Eck, N. J. From Louvain to Leiden: guaranteeing well-connected communities. *Sci. Rep.* **9**, 5233 (2019).
3. Lohoff, T. *et al.* Integration of spatial and single-cell transcriptomic data elucidates mouse

organogenesis. *Nat. Biotechnol.* 1–12 (2021).

4. Jackson, H. W. *et al.* The single-cell pathology landscape of breast cancer. *Nature* **578**, 615–620 (2020).
5. Moffitt, J. R. *et al.* Molecular, spatial, and functional single-cell profiling of the hypothalamic preoptic region. *Science* **362**, (2018).
6. Hartmann, F. J. *et al.* Single-cell metabolic profiling of human cytotoxic T cells. *Nat. Biotechnol.* (2020) doi:10.1038/s41587-020-0651-8.
7. Hie, B., Bryson, B. & Berger, B. Efficient integration of heterogeneous single-cell transcriptomes using Scanorama. *Nat. Biotechnol.* **37**, 685–691 (2019).
8. Stickels, R. R. *et al.* Highly sensitive spatial transcriptomics at near-cellular resolution with Slide-seqV2. *Nat. Biotechnol.* (2020) doi:10.1038/s41587-020-0739-1.
9. Dries, R. *et al.* Giotto: a toolbox for integrative analysis and visualization of spatial expression data. *Genome Biol.* **22**, 78 (2021).
10. La Manno, G. *et al.* RNA velocity of single cells. *Nature* **560**, 494–498 (2018).
11. Paul, F. *et al.* Transcriptional Heterogeneity and Lineage Commitment in Myeloid Progenitors. *Cell* **163**, 1663–1677 (2015).
12. pbmc3k -Datasets -Single Cell Gene Expression -Official 10x Genomics Support. https://support.10xgenomics.com/single-cell-gene-expression/datasets/1.1.0/pbmc3k.
13. Anderson, A. & Lundeberg, J. sepal: Identifying Transcript Profiles with Spatial Patterns by Diffusion-based Modeling. *Bioinformatics* (2021) doi:10.1093/bioinformatics/btab164.
14. Biancalani, T. *et al.* Deep learning and alignment of spatially-resolved whole transcriptomes of single cells in the mouse brain with Tangram. 2020.08.29.272831 (2020) doi:10.1101/2020.08.29.272831.
15. Tasic, B. *et al.* Shared and distinct transcriptomic cell types across neocortical areas. *Nature* **563**, 72–78 (2018).
16. Pham, D. *et al.* stLearn: integrating spatial location, tissue morphology and gene expression to find cell types, cell-cell interactions and spatial trajectories within undissociated tissues. *Bioarxiv* (2020) doi:10.1101/2020.05.31.125658.

17. Butler, A., Hoffman, P., Smibert, P., Papalexi, E. & Satija, R. Integrating single-cell transcriptomic data across different conditions, technologies, and species. *Nat. Biotechnol.* **36**, 411–420 (2018).
18. Righelli, D. *et al.* SpatialExperiment: infrastructure for spatially resolved transcriptomics data in R using Bioconductor. *Cold Spring Harbor Laboratory* 2021.01.27.428431 (2021) doi:10.1101/2021.01.27.428431.
19. Bergenstråhle, J., Larsson, L. & Lundeberg, J. Seamless integration of image and molecular analysis for spatial transcriptomics workflows. *BMC Genomics* **21**, 482 (2020).
20. Solorzano, L., Partel, G. & Wählby, C. TissUUmaps: interactive visualization of large-scale spatial gene expression and tissue morphology data. *Bioinformatics* **36**, 4363–4365 (2020).
