# Supplementary material for: Squidpy: a scalable framework for spatial omics analysis
Source: Nat Methods. 2022 Jan 31;19(2):171–8. doi: 10.1038/s41592-021-01358-2 (PMC8828470; doi:10.1038/s41592-021-01358-2)
Supplement: Supplementary file 3 — Peer Review Information [file 41592_2021_1358_MOESM3_ESM.pdf]

natureresearch

Peer Review Information

Journal: Nature Methods
Manuscript Title: Squidpy: a scalable framework for spatial omics analysis
Corresponding author name(s): Fabian J. Theis

Reviewer Comments & Decisions:

Decision Letter, initial version:

Subject: Additional input - Decision on Nature Methods submission NMETH-BC45303
Message:

13th Apr 2021

Dear Professor Theis,

We would like to add some additional editorial input on the revision. To fully address the criticisms raised by Reviewers 2 and 3, we think substantial improvement of the tool (eg, to show Squidpy technology-agnostic and superior over existing methods) following our reviewers' suggestions is needed.

Thank you again.

Sincerely,

Lin

Lin Tang, PhD
Senior Editor
Nature Methods

****

Date: 13th Apr 21 21:45:18
Last Sent: 13th Apr 21 22:06:05

From: Lin.tang@nature.com
To: fabian.theis@helmholtz-muenchen.de
CC: methods@us.nature.com
Subject: Decision on Nature Methods submission NMETH-BC45303
Message: 13th Apr 2021

Dear Professor Theis,

Your Brief Communication, "Squidpy: a scalable framework for spatial single cell analysis", has now been seen by 3 reviewers. As you will see from their comments below, although the reviewers find your work of potential interest, they have raised a number of important concerns. We are interested in the possibility of publishing your paper in Nature Methods, but would like to consider your response to these concerns before we reach a final decision on publication.

We therefore invite you to revise your manuscript to address these concerns. If more space is needed, you could consider expand the paper as an Article.

We are committed to providing a fair and constructive peer-review process. Do not hesitate to contact us if there are specific requests from the reviewers that you believe are technically impossible or unlikely to yield a meaningful outcome.

When revising your paper:

* include a point-by-point response to the reviewers and to any editorial suggestions

* please underline/highlight any additions to the text or areas with other significant changes to facilitate review of the revised manuscript

* address the points listed described below to conform to our open science requirements

* ensure it complies with our general format requirements as set out in our guide to authors at www.nature.com/naturemethods

* resubmit all the necessary files electronically by using the link below to access your home page

[REDACTED]

Note: This URL links to your confidential home page and associated information about manuscripts you may have submitted, or that you are reviewing for us. If you wish to forward this email to co-authors, please delete the link to your homepage.

We hope to receive your revised paper within six weeks. We are very aware of the difficulties caused by the COVID-19 pandemic to the community. If you cannot send it within this time, please let us know. In this event, we will still be happy to reconsider your paper at a later date so long as nothing similar has been accepted for publication at Nature Methods or published elsewhere.

OPEN SCIENCE REQUIREMENTS

REPORTING SUMMARY AND EDITORIAL POLICY CHECKLISTS
When revising your manuscript, please update your reporting summary and editorial policy checklists.

Reporting summary: https://www.nature.com/documents/nr-reporting-summary.zip
Editorial policy checklist: https://www.nature.com/documents/nr-editorial-policy-checklist.zip

If your paper includes custom software, we also ask you to complete a supplemental reporting summary.

Software supplement: https://www.nature.com/documents/nr-software-policy.pdf

Please submit these with your revised manuscript. They will be available to reviewers to aid in their evaluation if the paper is re-reviewed. If you have any questions about the checklist, please see http://www.nature.com/authors/policies/availability.html or contact me.

Please note that these forms are dynamic 'smart pdfs' and must therefore be downloaded and completed in Adobe Reader. We will then flatten them for ease of use by the reviewers. If you would like to reference the guidance text as you complete the template, please access these flattened versions at http://www.nature.com/authors/policies/availability.html.

IMAGE INTEGRITY
When submitting the revised version of your manuscript, please pay close attention to Digital Image Integrity Guidelines and to the following points below:

-- that unprocessed scans are clearly labelled and match the gels and western blots presented in figures.
-- that control panels for gels and western blots are appropriately described as loading on sample processing controls

-- all images in the paper are checked for duplication of panels and for splicing of gel lanes.

Finally, please ensure that you retain unprocessed data and metadata files after publication, ideally archiving data in perpetuity, as these may be requested during the peer review and production process or after publication if any issues arise.

DATA AVAILABILITY
We strongly encourage you to deposit all new data associated with the paper in a persistent repository where they can be freely and enduringly accessed. We recommend submitting the data to discipline-specific and community-recognized repositories; a list of repositories is provided here:
http://www.nature.com/sdata/policies/repositories

All novel DNA and RNA sequencing data, protein sequences, genetic polymorphisms, linked genotype and phenotype data, gene expression data, macromolecular structures, and proteomics data must be deposited in a publicly accessible database, and accession codes and associated hyperlinks must be provided in the “Data Availability” section.

Refer to our data policies here: https://www.nature.com/nature-research/editorial-policies/reporting-standards#availability-of-data

To further increase transparency, we encourage you to provide, in tabular form, the data underlying the graphical representations used in your figures. This is in addition to our data-deposition policy for specific types of experiments and large datasets. For readers, the source data will be made accessible directly from the figure legend. Spreadsheets can be submitted in .xls, .xlsx or .csv formats. Only one (1) file per figure is permitted: thus if there is a multi-paneled figure the source data for each panel should be clearly labeled in the csv/Excel file; alternately the data for a figure can be included in multiple, clearly labeled sheets in an Excel file. File sizes of up to 30 MB are permitted. When submitting source data files with your manuscript please select the Source Data file type and use the Title field in the File Description tab to indicate which figure the source data pertains to.

Please include a “Data availability” subsection in the Online Methods. This section should inform readers about the availability of the data used to support the conclusions of your study, including accession codes to public repositories, references to source data that may be published alongside the paper, unique identifiers such as URLs to data repository entries, or data set DOIs, and any other statement about data availability. At a minimum, you should include the following statement: “The data that support the findings of this study are available from the corresponding author upon request”, describing which data is available upon request and mentioning any restrictions on availability. If DOIs are provided, please include these in the Reference list (authors, title, publisher (repository name),

identifier, year). For more guidance on how to write this section please see:
http://www.nature.com/authors/policies/data/data-availability-statements-data-citations.pdf

CODE AVAILABILITY
Please include a “Code Availability” subsection in the Online Methods which details how your custom code is made available. Only in rare cases (where code is not central to the main conclusions of the paper) is the statement “available upon request” allowed (and reasons should be specified).

We request that you deposit code in a DOI-minting repository such as Zenodo, Gigantum or Code Ocean and cite the DOI in the Reference list. We also request that you use code versioning and provide a license.

For more information on our code sharing policy and requirements, please see:
https://www.nature.com/nature-research/editorial-policies/reporting-standards#availability-of-computer-code

MATERIALS AVAILABILITY
As a condition of publication in Nature Methods, authors are required to make unique materials promptly available to others without undue qualifications.

Authors reporting new chemical compounds must provide chemical structure, synthesis and characterization details. Authors reporting mutant strains and cell lines are strongly encouraged to use established public repositories.

More details about our materials availability policy can be found at https://www.nature.com/nature-portfolio/editorial-policies/reporting-standards#availability-of-materials

ORCID
Nature Methods is committed to improving transparency in authorship. As part of our efforts in this direction, we are now requesting that all authors identified as ‘corresponding author’ on published papers create and link their Open Researcher and Contributor Identifier (ORCID) with their account on the Manuscript Tracking System (MTS), prior to acceptance. This applies to primary research papers only. ORCID helps the scientific community achieve unambiguous attribution of all scholarly contributions. You can create and link your ORCID from the home page of the MTS by clicking on ‘Modify my Springer Nature account’. For more information please visit please www.springernature.com/orcid.

Please do not hesitate to contact me if you have any questions or would like to discuss these revisions further. We look forward to seeing the revised manuscript and thank you for the opportunity to consider your work.

Sincerely,

Lin

Lin Tang, PhD
Senior Editor
Nature Methods

Reviewers' Comments:

Reviewer #1:
Remarks to the Author:
I think this is nice paper that describes a potentially impactful framework for spatial data analysis. While still in its infancy, spatial technologies (and associated computational analyses) are poised to become routine for understanding and characterizing tissue architectures.

The overall framework seems fairly well-built interfacing with state-of-the-art imaging and single-cell framework (including scanpy) in Python.

I have a few comments below that I think will improve the manuscript:

1) Overall flow. I find the manuscript a bit difficult to read jumping from one example (and one data type) to the next. I think it would better for the authors to focus on a few use cases and create complete workflows for each use case. Then the components of each analysis workflow could be linked and referenced through the different sections. The workflows could then be made available online to support the paper. See for example: https://bioconductor.org/books/release/OSCA/ that came with the following paper
Amezquita, R.A., Lun, A.T.L., Becht, E., Carey, V.J., Carpp, L.N., Geistlinger, L., Marini, F., Rue-Albrecht, K., Risso, D., Soneson, C., Waldron, L., Pagès, H., Smith, M.L., Huber, W., Morgan, M., Gottardo, R., Hicks, S.C., 2020. Orchestrating single-cell analysis with Bioconductor. Nat. Methods 17, 137–145.

2) Title. The title says "single-cell" but not all technologies are single-cell, so perhaps drop single-cell from the title.

Other more minor comments:

I wouldn't call Tangram and cell2location deconvolution methods, although they can be used for that. They are more like alignment methods.

Another approach for spatial enhancement that doesn't require single-cell data is BayesSpace: https://www.biorxiv.org/content/10.1101/2020.09.04.283812v1

Finally, can SpatialExperiment objects (from Bioconductor) be imported in squidpy?

Reviewer #2:
Remarks to the Author:
The paper presents a framework, rather than a tool, for developers that work with analysis of spatial omics data. It builds on a very large number of python libraries, with the lab's own tools for single cell data (Scanpy) and data representation using (Anndata) in focus, but also connects to other python-based tools used within the community. A number of examples of how the framework can be used are presented, and accompanied by ready-to-run tutorials with data and on line.

A number of frameworks similar to Squidpy are available. The most similar framework is the recently published Giotto (https://pubmed.ncbi.nlm.nih.gov/33685491/); a very powerful tool for both spatial analytics and visualization based on a combination of javascript, R, and python. This tool does however not provide methods for image analysis, such as cell segmentation and extraction of features from tissue morphology.

Also the computational framework Starfish (https://spacetx-starfish.readthedocs.io/en/stable/), created within the HumanCellAtlas consortium, has some overlap with Squidpy. Starfish is a Python library for generating gene expression matrices from image-based spatially-resolved transcriptomics assays. Starfish is so far primarily focused on decoding raw data, including e.g. cell segmentation, while not performing spatial statistics.

For fast and efficient visualization, web-based tools such as TissUUmaps (https://doi.org/10.1093/bioinformatics/btaa541) have the great benefit that they allow multi-user interactive viewing and interaction without the requirement of the user having to download large amounts of image data, as is the case with Squidpy, but it does not perform data analysis.

Further related tools are nicely presented in table 1 of the paper, and with its wide compatibility Squidpy does indeed cover more types of analysis for 2D spatial omics data than other available tools.

The modularity makes Squidpy flexible, but also quite scattered, and it is likely most valuable for developers while not very easy to navigate for a python beginner. At the same time, if gaining sufficient momentum in the fast-evolving spatial omics field, it has the potential to become a widely used framework. The authors should be credited for the availability of a number of tutorials related both to data and figures in the manuscript, but also beyond this.

The main question regarding this manuscript falls back to novelty. None of the methods introduced are novel, and many of the methods presented in the results section, such as the cell segmentation and the spatial statistics tools, are not state-of-the art (e.g. for cell segmentation the user is referred to using learning-based methods, but examples are not provided). Even bridging to tools such as CellProfiler (cellprofiler.org) for cell feature extraction would be interesting here.

There is a large number of popular spatial statistics tools in R (e.g. rspatial.org) that are far more powerful than the methods presented in Squidpy (that e.g. do not manage to find the elongated T-cell- and endothelial-cell-rich structures in Fig 3a). For Squidpy to become fully powerful in its attempt to solve spatial statistics questions it should also bridging to these methods using rpy2 (https://pypi.org/project/rpy2/).

The paper is well-written, and when complemented with the on-line data and tutorials and supplementary information the presentation should be regarded as being of high quality.

A minor concern is with the integration with Tangram, which is interesting, but not very convincing, and supplementary figure 4 requires a more in-depth discussion and explanation. It would be more convincing to also illustrate Tangram integration with a larger dataset such as https://www.cell.com/cell/pdf/S0092-8674(19)31282-6.pdf

Minor comments:
line 44, add plural: ‘enables community-driven scalable analysis of both spatial neighborhood graphs and images, ‘

Squidpy handles large images by tiling: Edge effects at tiling prior to cell segmentation and extraction of cell and image features should be presented/discussed.

Reviewer #3:
Remarks to the Author:
Palla et al presents squidpy, an package for analyzing spatial transcriptomic data:

Significance: accessible and efficient software packages are needed to analyze and visualize various kinds of data that are rapidly becoming available via new spatial transcriptomics techniques. However, the software ecosystem for analyzing and visualizing these datasets across techniques and modalities is fragmented. Palla et al. provide a software package enabling efficient analysis of spatial gene expression graphs and image data, as well as describing potentially transformative approaches for joint analysis of molecular and morphological information.

Approach: The authors build their software package around two data structures, a neighborhood graph encoding spatial proximity, and an image container object for working with tissue images. The authors further provide a library of methods for computing spatial statistics on the neighborhood graph, and methods for image processing, along with interfaces to external tools. The authors validate their neighborhood graph approach against existing datasets, show its applicability to multiple different experimental techniques by quantifying several properties of tissue organization, and demonstrate joint analysis of spatial gene expression and image morphology.

Results: Technically, neighborhood enrichment, visualization, cluster co-occurrence, spatial variability detection, ligand-receptor interaction detection, DAPI segmentation, and H&E stain clustering are properly executed. The authors observe some agreement with published datasets in their neighborhood enrichment analysis (but see minor comments), and their visualizations and tissue property quantifications are biologically plausible. Their image segmentation pipeline is sound. They demonstrate the technical feasibility of applying Squidpy for joint analysis of molecular data and morphology.

Major points:

1) A key claim of the manuscript is that Squidpy bridges spatial graph analysis methods and image analysis methods, offering joint analysis of both molecular and morphological data. This has the potential to be transformative, and it is exciting to think about the types of analyses such an approach could enable. However, the demonstrations presented make it difficult to assess the likely impact of Squidpy's approach. The DAPI segmentation is technically sound, but does not take advantage of the spatial graph methods central to Squidpy, and could have been carried out with existing packages (e.g. stLearn). The H&E stain analysis, on the other hand, does take advantage of the spatial graph, and has the potential to deliver a powerful demonstration of a joint molecular/morphology approach. Unfortunately, their comparison of summary image features with gene clusters is not very illuminating --

for instance, can the authors offer a biological interpretation for what the H&E feature clusters are, or why they overlap with the hippocampus, but not the cortex? If this type of joint analysis is the main advance put forward by the paper, it would be valuable to demonstrate at least one biologically interpretable and relevant application that would be really motivating, to make someone want to install and use this package.

2) A central claim of the manuscript is that Squidpy is technology-agnostic, and in support of this claim the authors demonstrate their approach across four different technologies: Visium, SeqFISH, 4i, and Imaging Mass Cytometry. This is a thoughtful and impressive demonstration which shows Squidpy can be used with spatial technologies which are spot-based and molecular imaging-based. However, the discussion of these modalities is incomplete, with the authors failing to mention higher-resolution bead-array modalities for spatial transcriptomics (e.g. HDST, Slide-Seq) and thick-sample 3D modalities for molecular imaging (e.g. merFISH, ISS, STARmap). The manuscript would greatly benefit from a considered and careful discussion of these modalities, and the manuscript should offer greater evidence supporting the claim of technology agnosticism by including these modalities or else significantly moderate its claims (e.g. it is not obvious from the manuscript Squidpy is capable of working with, say, 3D data).

3) The authors explicitly position their software package as an alternative to the existing Giotto package. The authors of Giotto demonstrated their package across nine different technologies versus Squidpy's four, including the bead (Slideseq) and 3D (merFISH, STARmap) modalities discussed above. Palla et al. benchmark Squidpy against Giotto, showing a claimed 10-fold speed-up and concluding "Squidpy outperforms similar functions provided by the Giotto toolkit, for any dataset and task" (Fig S1). This speedup is an outstanding achievement of which the authors should be very proud. However, as discussed above, it is not clear to me that Squidpy is compatible with all of the data modalities analyzed by Giotto -- e.g. what is the benchmark for dataset = "STARmap 3D visual cortex"? The manuscript would greatly benefit from a fair and nuanced comparison of these two packages across all relevant modalities/datasets, with appropriate evidence provided when making such strong claims; otherwise these claims should be significantly moderated.

Suggestions: 1) show a clear use case and compelling biological demonstration for spatial graphs + images, 2) show Squidpy is applicable to a wider range of techniques or significantly moderate claims 3) carefully re-work sections of the manuscript to make a more a nuanced and fair comparison to existing methods.

Minor points:

1) Fig 2a, shows “lateral plate mesoderm” is co-enriched with "allantois" and "intermediate mesoderm", and “endothelium’ is enriched with “haematoendothelial progenitors”, and the authors point out this matches the results of Lohoff et al. However, sticking with this example, “haematoendothelial progenitors” are co-enriched with "allantois", "intermediate mesoderm", and "lateral plate mesoderm" in Lohoff et al, and this result is not evident in the analysis of Palla et al. While it seems by visual inspection that there are many commonalities between these two sets of results, it would inspire greater confidence if the manuscript included a more systematic comparison of these two sets of results, with explanations provided for discrepancies arising, and rationales given for why these specific comparisons were drawn while others were omitted.

2) Although the claimed ~10-fold speedup vs. Giotto is, as mentioned, quite impressive, visual inspection of Fig S1 suggests that this might be somewhat overstated, with only the 4i dataset definitively showing a speedup of this magnitude by visual inspection. The authors may want to annotate this chart to provide more obvious evidence for their claim. It is also unclear whether this large speedup applies across the full suite of functionality in Giotto (e.g. visualization) or specifically to graph-building and neighborhood enrichment only.

3) It would be helpful if the authors can describe how they pre-process their datasets in the methods section.

4) Spelling error in Fig 2g (‘nucleous’)

Author Rebuttal to Initial comments

Point-by-point response to the reviewers’ comments

Title: Squidpy: a scalable framework for spatial omics analysis

Authors: Giovanni Palla*1,2, Hannah Spitzer*1, Michal Klein1, David Fischer1,2, Anna Christina Schaar1,2, Louis Benedikt Kuemmerle1,4, Sergei Rybakov1,3, Ignacio L. Ibarra1, Olle Holmberg1, Isaac Virshup5, Mohammad Lotfollahi1,2, Sabrina Richter1,2, Fabian J. Theis1,2,3+

1 Institute of Computational Biology, Helmholtz Center Munich, Germany.
2 TUM School of Life Sciences Weihenstephan, Technical University of Munich, Germany.3 Department of Mathematics, Technical University of Munich, Germany.
4 Institute for Tissue Engineering and Regenerative Medicine (iTERM), Helmholtz Center Munich, Germany

5 Department of Anatomy and Physiology, University of Melbourne, Australia.

*equal contribution
+Correspondence: fabian.theis@helmholtz-muenchen.de

In the following, we present our response to the reviewers comments. We give comments (black), point-by-point answers (green) to the questions and in parts copy parts of the text or specific panels (beige), which directly correspond to comments or reference to them.

Reviewer #1 (Remarks to the Author):

I think this is nice paper that describes a potentially impactful framework for spatial data analysis. While still in its infancy, spatial technologies (and associated computational analyses) are poised to become routine for understanding and characterizing tissue architectures.
The overall framework seems fairly well-built interfacing with state-of-the-art imaging and single-cell framework (including scanpy) in Python.

We would like to thank the reviewer for the positive assessment. Below, we address each comment individually.

Major comments:

1) Overall flow. I find the manuscript a bit difficult to read jumping from one example (and one data type) to the next. I think it would better for the authors to focus on a few use cases and create complete workflows for each use case. Then the components of each analysis workflow could be linked and referenced through the different sections. The workflows could then be made available online to support the paper. See for example: https://bioconductor.org/books/release/OSCA/ that came with the following paper
Amezquita, R.A., Lun, A.T.L., Becht, E., Carey, V.J., Carpp, L.N., Geistlinger, L., Marini, F., Rue-Albrecht, K., Risso, D., Soneson, C., Waldron, L., Pagès, H., Smith, M.L., Huber, W., Morgan, M., Gottardo, R., Hicks, S.C., 2020. Orchestrating single-cell analysis with Bioconductor. Nat. Methods 17, 137–145.

We would like to thank the reviewer for the constructive feedback. As suggested, we re-structured the manuscriptin order to extend the description of Squidpy's functionality and to improve the fragmented text, and we added an exemplary analysis workflow on spatial transcriptomics data. We worked out in detail 7 data-centric workflows and use-cases, but to avoid repetition only included a single one (Visium) in the text. The "Result" section is nowdivided in 3 main paragraphs:

- *Squidpy provides infrastructure and analysis tools to identify spatial patterns in tissue*, where we are describing Squidpy’s capabilities in spatial graph building and analysis tools for mining spatial patterns in tissue, for continuous and discrete covariates.

- *Squidpy enables analysis and visualization of large images in spatial omics data*, where we describe Squidpy's infrastructure and analysis tools for the large tissue images often acquired in spatial omics data (both transcriptomics and proteomics).
- *Squidpy's workflow enables the integrative analysis of spatial transcriptomics data*, where we show an exemplary analysis workflow for 10X genomics Visium spatial transcriptomics data of the Mouse brain coronal section.

We think that this separation highlights both Squidpy's technology-agnostic capabilities (paragraph 1 and 2) but also provides insights into how the functionalities can be tied together to fully leverage the new modalities in spatial omics data and gain new insights from the data (paragraph 3).

As mentioned already, we want to highlight that Squidpy's documentation (which can be found at https://squidpy.readthedocs.io) already provides a very rich set of examples and workflows that closely mirror the scope of the above mentioned OSCA resource (and we are aware of the building of OSTA for spatial data: https://lmweber.org/OSTA-book/). In particular, we categorize 3 types of resources:

- Examples (https://squidpy.readthedocs.io/en/latest/examples.html): short and concise examples of specific Squidpy functionalities.
- Core tutorials (https://squidpy.readthedocs.io/en/latest/tutorials.html#core-tutorials): tutorial workflows that showcase how Squidpy functionalities can be tied together to analyze specific dataset (shown for Visium, IMC, MERFISH, seqFISH, SlideseqV2, 4i and Mibi-tof datasets), as well as specific methods that require an extended explanation (e.g. Napari).
- External tutorials (https://squidpy.readthedocs.io/en/latest/tutorials.html#external-tutorials): tutorials on the usage of external tools with Squidpy, such as segmentation methods.
    - StarDist: https://squidpy.readthedocs.io/en/latest/external_tutorials/tutorial_stardist.html
    - Cellpose: https://squidpy.readthedocs.io/en/latest/external_tutorials/tutorial_cellpose_segmentation.html
    - Tangram: https://squidpy.readthedocs.io/en/latest/external_tutorials/tutorial_tangram.html
    - CellProfiler: https://squidpy.readthedocs.io/en/latest/external_tutorials/tutorial_cellprofiler.html
    - Tensorflow: https://squidpy.readthedocs.io/en/latest/external_tutorials/tutorial_tf.html
    - Napari: https://squidpy.readthedocs.io/en/latest/external_tutorials/tutorial_napari.html

All these resources are tightly linked together and with the Squidpy API, for instance, the tutorial on 10x genomics Visium H&E data (https://squidpy.readthedocs.io/en/latest/auto_tutorials/tutorial_visium_fluo.html) showcases the spatial graph building method linking both the example (https://squidpy.readthedocs.io/en/latest/auto_examples/graph/compute_spatial_neighbors.html#sphx-glr-auto-examples-graph-compute-spatial-neighbors-py), and the method API (https://squidpy.readthedocs.io/en/latest/api/squidpy.gr.spatial_neighbors.html#squidpy.gr.spatial_neighbors), andin the API other examples and tutorials that use the same method can be found.
We also want to point out that "Examples" (https://squidpy.readthedocs.io/en/latest/examples.html) and "Core tutorials" (https://squidpy.readthedocs.io/en/latest/tutorials.html) are incorporated in the Continuous Integration pipeline (https://github.com/theislab/squidpy_notebooks/actions), so they are thoroughly tested and any change in the Squidpy repository that introduces breaks or changes existing functionalities will be promptly identified (we

added this comment in lines 64-65).
While we cover detailed analysis workflows in the new manuscript version and in the tutorial notebooks, we would like to point out that spatial omics workflows, much like single-cell workflows with Scanpy, are derived fromheavily branched pipelines which often result in very unique analysis scenarios for each individual project. A core contribution of Scanpy/Anndata in this field was that it enabled maintenance of an ecosystem that facilitates building and extending such highly customized workflows. We view Squidpy as the software equivalent for spatial omics data and did therefore try to also highlight this point in the current version of the manuscript, that Squidpy is not just a series of workflows for each different spatial data modality, but a framework that allows mix-and-match workflows across data modalities. Finally, we would like to acknowledge that spatial omics data analysis is a nascent field and therefore there are few established best practices. We hope with Squidpy to spark the interest in developing those and build a community of users and tools that is tightly integrated in the single cell analysis ecosystem in Python. We included a short mention of this discussion in the manuscript in order to acknowledge the fact that this field is still nascent (lines 252-254).

2) Title. The title says "single-cell" but not all technologies are single-cell, so perhaps drop single-cell from the title.

We thank the reviewer for the comment. Following up on this, we had a long internal discussion on the scale of applicability of Squidpy, and ended up agreeing to this comment eventually. We acknowledge that spatial omics technologies provide a varying degree of resolution, and therefore single-cell might be inappropriate. We therefore changed the title to: "Squidpy: a scalable framework for spatial omics analysis."

Minor comments:
I wouldn't call Tangram and cell2location deconvolution methods, although they can be used for that. They are more like alignment methods.

We thank the reviewer for the suggestion. Indeed in math we would prefer to call those algorithms "decomposition", since they need a basis of singletons. However, we would like to point out that the task of determining proportions of cell types in mixed samples is nowadays commonly referred to as "deconvolution" (see e.g. a recent benchmark for bulk RNA-seq [1] or methods tailored for spatial data that explicit use the term deconvolution in the title [2–4]). While decomposition would be an alternative term (e.g. [5]), the term "deconvolution"is quite more prevalent. We think that "alignment" is a more appropriate term for data integration approaches, although it is true that data integration methods can be used for deconvolution approaches (e.g. Seurat or Scanorama in Scanpy). We added this in brackets to make sure readers see we made a choice here (line 158): In addition, we can leverage segmented nuclei to inform cell-type deconvolution (or decomposition/mapping) methods such as Tangram or Cell2Location.

Another approach for spatial enhancement that doesn't require single-cell data is BayesSpace:
https://www.biorxiv.org/content/10.1101/2020.09.04.283812v1

We thank the reviewers for pointing to this method. BayesSpace is a method for spatial transcriptomic clustering that uses a t-distributed error model to identify spatial clusters. It is useful for specific tasks such as high-resolution gene expression mapping and clustering. We added a citation at line 42.

Finally, can SpatialExperiment objects (from Bionconductor) be imported in squidpy?

SpatialExperiment is a R-Bioconductor object to store spatial omics data. It is an S4 class based on the SummarizedExperiment object [6]. We attempted to convert spatial omics Anndata objects into SingleCellExperiment objects with Zellkonverter (official bioconductor tool to convert between scRNAseq object across platforms: https://bioconductor.org/packages/release/bioc/html/zellkonverter.html ,attempted conversion can be found here https://github.com/theislab/squidpy_reproducibility/blob/master/notebooks/reproducibility/spatialExperiment_convert.R). However, we were not successful. The converted object only retained the "molecular" level information without the "spatial" information (spatial coordinates and the small low-resolution tissue image). We would also like to acknowledge that the SpatialExeriment object does neither store nor provide methods to analyze the largetissue image (which is stored in the ImageContainer in Squidpy). Until such data can be stored in a Bioconductorobject, we will not be able to fully convert data and analysis results between the two platforms. For the moment, functionality of SpatialExperiment is still too limited and since the package is still being actively developed, this results in frequent changes to the interface. We will therefore wait for further development before implementing a stable object conversion between Anndata and SpatialExperiment together with the SpatialExperiment developeras well as developers of current frameworks of object-conversion methods (Zellkonverter, Anndata2ri).

Reviewer #2 (Remarks to the Author):

The paper presents a framework, rather than a tool, for developers that work with analysis of spatial omics data. It builds on a very large number of python libraries, with the lab's own tools for single cell data (Scanpy) and data representation using (Anndata) in focus, but also connects to other python-based tools used within the community. A number of examples of how the framework can be used are presented, and accompanied by ready-to-run tutorials with data and on line.

A number of frameworks similar to Squidpy are available. The most similar framework is the recently published Giotto (https://pubmed.ncbi.nlm.nih.gov/33685491/); a very powerful tool for both spatial analytics and visualization based on a combination of javascript, R, and python. This tool does however not provide methodsfor image analysis, such as cell segmentation and extraction of features from tissue morphology.

Also the computational framework Starfish (https://spacetx-starfish.readthedocs.io/en/stable/), created within the HumanCellAtlas consortium, has some overlap with Squidpy. Starfish is a Python library for generating gene expression matrices from image-based spatially-resolved transcriptomics assays. Starfish is so far primarily focused on decoding raw data, including e.g. cell segmentation, while not performing spatial statistics.

For fast and efficient visualization, web-based tools such as TissUUmaps (https://doi.org/10.1093/bioinformatics/btaa541) have the great benefit that they allow multi-user interactive viewing and interaction without the requirement of the user having to download large amounts of image data, asis the case with Squidpy, but it does not perform data analysis.

Further related tools are nicely presented in table 1 of the paper, and with its wide compatibility Squidpy doesindeed cover more types of analysis for 2D spatial omics data than other available tools.

We thank the reviewer for this positive evaluation of the manuscript and the overview over related packages. Note that we have added an extensive comparison with Giotto in Supplementary Table 2.
Thank you for the link to TissUUmaps, this is a very useful tool for interactive exploration of spatial data, and we added it to Supplementary Table 1 and cited it. We now also explicitly mentioned Starfish in line 41 to clarify that Squidpy, amongst others, is meant to be a framework for analysis methods applied on processed data that are e.g. the result of Starfish workflows.

Major comments:
The modularity makes Squidpy flexible, but also quite scattered, and it is likely most valuable for developers while not very easy to navigate for a python beginner. At the same time, if gaining sufficient momentum in the fast-evolving spatial omics field, it has the potential to become a widely used framework. The authors should be credited for the availability of a number of tutorials related both to data and figures in the manuscript, but alsobeyond this.

Thank you for recognising the modularity of Squidpy; making this package flexible and easily extendable was oneof the main design goals during the development. We use object-oriented design, refactoring principles, and havean extensive testing suite using Continuous Integration to ensure high code quality. While it is correct that these principles might make *developing core Squidpy code* difficult for python beginners, they ensure that Squidpy is flexible, and can be easily adapted to new analysis methods. Its modularity is also key to make it easy to interface with the other tools of the single cell analysis ecosystem in Python. However, despite these powerful abstractions in the core code, the package provides an intuitive functional API, following the Scanpy API, that was designed to make Squidpy *easy to use for python beginners.* We have seen this working out very well (actually well beyond our imagination with more than 250k downloads) for Scanpy, with non-python users essentially just building upon existing notebooks. To enhance accessibility, we provide and now have extended an extensive documentation of each function, with usage-examples linked at the bottom of each page: https://squidpy.readthedocs.io/en/latest/api.html . As already noted, we also provide exemplary analysis tutorials for a diverse set of spatial technologies, which provides an ideal entry point for a python beginner: https://squidpy.readthedocs.io/en/latest/tutorials.html .
We have added clarification in the text with respect to this comment at lines 64-65:
It [Squidpy] provides a rich documentation, with tutorials and example workflows, integrated in the continuous integration pipeline.
and lines 246-248:

Squidpy's rich documentation in the form of functional API documentation, examples and tutorial workflows, is easy to navigate and accessible to both experienced developers and beginner analysts.

To also reflect easier access in the text, we now added a more detailed workflow example/use case; for an extended explanation of the workflow resources present in Squidpy, please see response to Reviewer #1 Major comment 1.

The main question regarding this manuscript falls back to novelty. None of the methods introduced are novel, and many of the methods presented in the results section, such as the cell segmentation and the spatial statistics tools, are not state-of-the art (e.g. for cell segmentation the user is referred to using learning-based methods, but examples are not provided). Even bridging to tools such as CellProfiler (cellprofiler.org) for cell feature extraction would be interesting here.

The main novelty of Squidpy is to provide a unified framework for analysis of diverse spatial omics datasets by combining analysis of spatial molecular information and image information and allowing for interactive visualisation, which has not been done before. By integrating and in parts making existing statistical methods accessible to the user, we allow integrated analyses far beyond what has been possible so far. The scalability and integration of analysis workflows in Squidpy enables qualitatively improved analyses in real-life settings withtime and computational resource constraints. Furthermore, we want to stress that Squidpy is designed in a flexible manner to allow an easy interface with novel analysis methods within the same framework.
To further strengthen this claim, we have extended the manuscript and online tutorials to include examples of using the state-of-the art nuclei segmentation methods such as Cellpose[7] and StarDist[8] within the Squidpy framework. We also provide an example of how to use CellProfiler[9] features with downstream analysis methods. The new Supplementary Fig. 5 shows these examples, which are also available as tutorials in the Squidpy documentation:

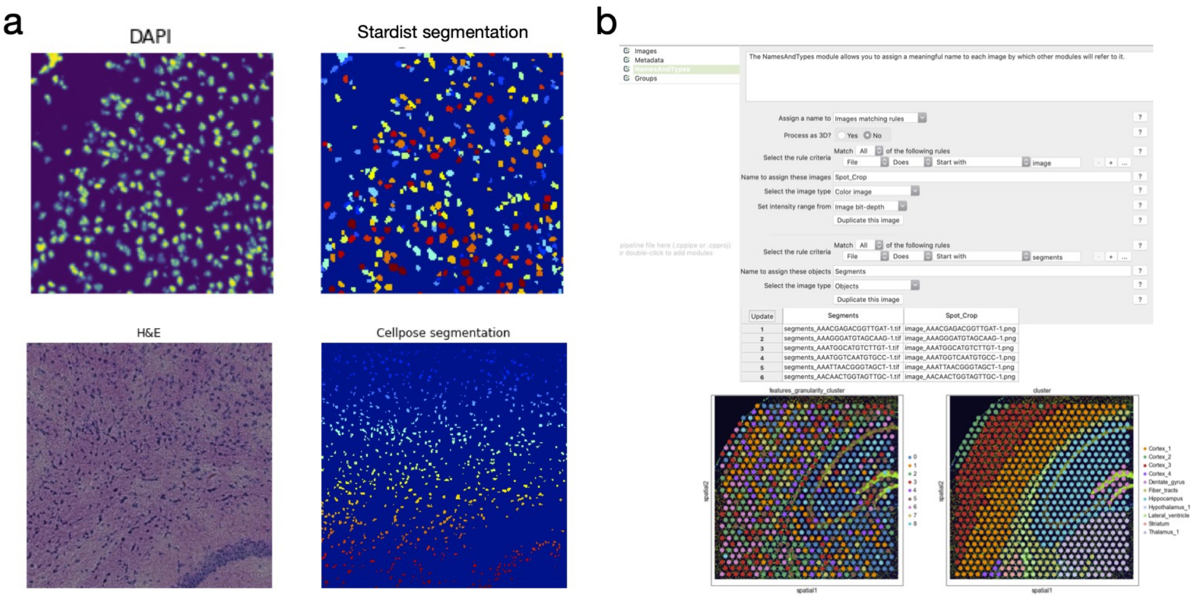

Supplementary Figure 5. Interfacing Squidpy to DL-based segmentation methods and CellProfiler.
(a) Segmentation results for DAPI staining with StarDist (top) and for H&E staining with Cellpose (bottom). Squidpy's Image Container is flexible to be interfaced with modern DL-based models. Both of these examples are part of the Squidpy documentation (see link in code and data availability sections).
(b) Example of CellProfiler pipeline interfaced with Squidpy: here, the CellProfiler pipeline performs segmentation and computes segmentation-masks features such as granularity, which is then used downstream by Squidpy to obtain cluster annotation (bottom-left clusters, compared to gene-clusters in bottom-right of the figure). This example is part of the Squidpy documentation (see https://squidpy.readthedocs.io/en/stable/external_tutorials/tutorial_cellpose_segmentation.htmll, https://squidpy.readthedocs.io/en/stable/external_tutorials/tutorial_stardist.html, and https://squidpy.readthedocs.io/en/stable/external_tutorials/tutorial_cellprofiler.html).

We additionally provide a tutorial on interfacing the Image Container with custom deep learning models using Tensorflow (ref. Supplementary Fig. 3).
The in-depth tutorials for the aforementioned extensions can be found here:

- StarDist: https://squidpy.readthedocs.io/en/latest/external_tutorials/tutorial_stardist.html
- Cellpose: https://squidpy.readthedocs.io/en/latest/external_tutorials/tutorial_cellpose_segmentation.html
- Tangram: https://squidpy.readthedocs.io/en/latest/external_tutorials/tutorial_tangram.html
- CellProfiler: https://squidpy.readthedocs.io/en/latest/external_tutorials/tutorial_cellprofiler.html
- Tensorflow: https://squidpy.readthedocs.io/en/latest/external_tutorials/tutorial_tf.html
- Napari: https://squidpy.readthedocs.io/en/latest/external_tutorials/tutorial_napari.html

Finally, although spatial statistics or ligand-receptor analysis methods are not novel from the statistical point of view, their implementations leveraging sparse arrays and Numba-compiled code are novel from the software-engineering side, thus making them very fast and able to scale to much larger datasets than before (see

Supplementary Fig. 1 for comparison to other tools, such as CellphoneDB, Sepal and Giotto functions).

There is a large number of popular spatial statistics tools in R (e.g. rspatial.org) that are far more powerful than the methods presented in Squidpy (that e.g. do not manage to find the elongated T-cell-and endothelial-cell-rich structures in Fig 3a). For Squidpy to become fully powerful in its attempt to solve spatial statistics questions it should also bridging to these methods using rpy2 (https://pypi.org/project/rpy2/).

We thank the reviewer for the suggestion. Rspatial is an online resource to teach spatial data analysis in R. However, the core package that implements spatial statistics is spatstat (https://spatstat.org/). Spatstat provides a rich set of tools and methods, mostly based on spatial point patterns, for the analysis of 2D spatial data, It is mostly focused on the analysis of geo-spatial data. It does not support data formats and infrastructure for handling spatial molecular data, thus making it very difficult to wrangle dataset shapes and formats from Squidpy to spatstat. Interestingly, even the R-based Giotto does not use this package, most probably due to similar reasons.

In addition, the challenge of converting data formats between platforms (Python to R) makes it complicated for developers to maintain and thoroughly test functions supported in Squidpy (as it is done now) and also can be complex for users, despite the availability of the rpy2 interface (which can be challenging unless paired with docker support). We therefore do not plan to support rpy2 and bridge Squidpy infrastructure to the spatstat package.

However, in the future, we will extend the support for spatial data analysis functions levearing implementations from pysal (Python Spatial Analysis Library https://github.com/pysal?type=source, a python equivalent to spatstat). In fact, in Squidpy 1.0.0, the method to calculate Moran's I spatial autocorrelation statistics was utilisingthe libpysal implementation in the backend. However, in Squidpy 1.1.0 we replaced the libpysal implementation with our own implementation using Numba (faster and supporting sparse arrays, method API at https://squidpy.readthedocs.io/en/latest/api/squidpy.gr.spatial_autocorr.html).

We anticipate to leverage other functions from libpysal in the future.

Finally, we extended the functionalities of Squidpy to compute spatial statistics, in particular, we added:

- Geary's C autocorrelation statistics (orthogonal to Moran's I): https://squidpy.readthedocs.io/en/latest/api/squidpy.gr.spatial_autocorr.html
- Sepal for spatially variable genes identification: https://squidpy.readthedocs.io/en/latest/api/squidpy.gr.sepal.html
- We implemented a variance-stabilized transformation for Ripley's K (Ripley's L) as well as RIpley's F and G functions: https://squidpy.readthedocs.io/en/latest/api/squidpy.gr.ripley.html

Regarding the comment:

that e.g. do not manage to find the elongated T-cell-and endothelial-cell-rich structures in Fig 3a

We are not sure what this comment is referring to. If the comments refers to Supplementary Fig. 3a (now Supplementary Fig. 2a), the enrichment between the "elongated" structures is in fact present in the neighborhood enrichment plot in Supplementary Fig. 2d (see bottom-left cluster with T-cells, elongated stromal cells, endothelial and macrophages cluster). This group of cell-types clearly clusters together in contrast to the rest of the

annotations, as can be also visualized in the dendrogram on top of the heatmap.

The paper is well-written, and when complemented with the on-line data and tutorials and supplementary information the presentation should be regarded as being of high quality.

Thank you for this positive assessment. We added a sentence to the introduction to make the availability of documentation and tutorials even clearer to the reader at lines 67-69.

A minor concern is with the integration with Tangram, which is interesting, but not very convincing, and supplementary figure 4 requires a more in-depth discussion and explanation. It would be more convincing to also illustrate Tangram integration with a larger dataset such as https://www.cell.com/cell/pdf/S0092-8674(19)31282-6.pdf

We thank the reviewer for the comment. The purpose of the Tangram integration is to showcase how Squidpy's image segmentation capability can complement Tangram's ability to leverage prior information on cell density for the deconvolution task. The advantages for the users are twofold: first, prior information on nuclei density under each spot can be useful for the deconvolution task performed by Tangram and second, the inferred cell types mapped to the segmentation objects coordinates can be easily visualized, either statically or with the Napari GUI. This is possible because the Image Container in Squidpy provides both segmentation capabilities as well as retains a unique mapping between single segmentation objects and Visium spots' area and coordinates. We extended the caption of Supplementary Fig. 4 to make this more clear.
We are not convinced that showcasing an additional Tangram example with the suggested dataset can enhance the paper, and have the following concerns:

- The images obtained by the spatial transcriptomics experiment[10] are H&E stained. Nuclei segmentation in H&E is less reliable and we would require extensive fine tuning to increase accuracy.
- The Spatial Transcriptomics experiments are not 10X Genomics Visium but the older generation of Spatial Transcriptomics technology. We are not aware that Tangram was applied to nor even designed for such data.
- The size of the data is large in the number of slides (19 slides) but for a single slide the number of observations (spots) is lower. Since this analysis needs to be performed for each slide, its application would not result in better insights on computation performances.
- The brain cortex example already provided is an easy to interpret and widely used system to showcase deconvolution results (see e.g. Seurat https://satijalab.org/seurat/articles/spatial_vignette.html or Scanpy https://scanpy-tutorials.readthedocs.io/en/latest/spatial/integration-scanorama.html integration tutorials)

What we did instead was to reach out to Tangram developers and discuss how to improve API design, ending upin designing an explicit API in Tangram to interface with Squidpy, thus shifting much code of our tutorial to their repository. We think this cleans up interfaces and makes the use case much clearer. The updated tutorial can be found at: https://squidpy.readthedocs.io/en/latest/external_tutorials/tutorial_tangram.html .

Minor comments:

line 44, add plural: ‘enables community-driven scalable analysis of both spatial neighborhood graphs and images
We corrected the sentence (now in line 45).

Squidpy handles large images by tiling: Edge effects at tiling prior to cell segmentation and extraction of cell and image features should be presented/discussed.

We would like to thank the reviewer for mentioning potential edge effects when using tiling to process images. We have now ported Squidpy's image analysis functions to use Dask Image[11], which has the advantage of usinglazy processing and accounting for edge effects by extracting overlapping image tiles (https://docs.dask.org/en/latest/array-overlap.html). Built-in processing and segmentation functions have default values for this overlap to minimize border effects. We added a sentence to the manuscript to make this clear at lines 145-146:
When using image tiling during processing, overlapping crops are used to mitigate border effects.

In addition, we have updated the usage-example for image processing in our documentation to discuss this parameter: https://squidpy.readthedocs.io/en/latest/auto_examples/image/compute_process_hires.html

---

Reviewer #3 (Remarks to the Author):
Palla et al presents squidpy, an package for analyzing spatial transcriptomic data:

Significance: accessible and efficient software packages are needed to analyze and visualize various kinds of data that are rapidly becoming available via new spatial transcriptomics techniques. However, the software ecosystem for analyzing and visualizing these datasets across techniques and modalities is fragmented. Palla etal. provide a software package enabling efficient analysis of spatial gene expression graphs and image data, as well as describing potentially transformative approaches for joint analysis of molecular and morphological information.

Approach: The authors build their software package around two data structures, a neighborhood graph encoding spatial proximity, and an image container object for working with tissue images. The authors further provide a library of methods for computing spatial statistics on the neighborhood graph, and methods for image processing, along with interfaces to external tools. The authors validate their neighborhood graph approach against existing datasets, show its applicability to multiple different experimental techniques by quantifying several properties of tissue organization, and demonstrate joint analysis of spatial gene expression and image morphology.

Results: Technically, neighborhood enrichment, visualization, cluster co-occurrence, spatial variability detection, ligand-receptor interaction detection, DAPI segmentation, and H&E stain clustering are properly executed. The authors observe some agreement with published datasets in their neighborhood enrichment analysis (but see

minor comments), and their visualizations and tissue property quantifications are biologically plausible. Their image segmentation pipeline is sound. They demonstrate the technical feasibility of applying Squidpy for joint analysis of molecular data and morphology.

We thank the reviewer for this positive evaluation. Below, we address each comment separately.

Major comments:

1) A key claim of the manuscript is that Squidpy bridges spatial graph analysis methods and image analysis methods, offering joint analysis of both molecular and morphological data. This has the potential to be transformative, and it is exciting to think about the types of analyses such an approach could enable. However, the demonstrations presented make it difficult to assess the likely impact of Squidpy's approach. The DAPI segmentation is technically sound, but does not take advantage of the spatial graph methods central to Squidpy, and could have been carried out with existing packages (e.g. stLearn). The H&E stain analysis, on the other hand, does take advantage of the spatial graph, and has the potential to deliver a powerful demonstration of a joint molecular/morphology approach. Unfortunately, their comparison of summary image features with gene clusters is not very illuminating -- for instance, can the authors offer a biological interpretation for what the H&E feature clusters are, or why they overlap with the hippocampus, but not the cortex? If this type of joint analysis is the main advance put forward by the paper, it would be valuable to demonstrate at least one biologically interpretable and relevant application that would be really motivating, to make someone want to install and use this package.

We would like to thank the reviewer for this very important comment. We agree that the main novelty of Squidpy lies in providing a unified framework for joint molecular and image data analysis. The main role of Squidpy is to facilitate this type of joint analysis and visualisation by leveraging core or external methods.
To more clearly show the additional insights that can be gained by analyzing both spatial patterns and the large tissue image, we restructured the manuscript and added a separate section on integrative analysis of spatial transcriptomics data (*Squidpy's workflow enables the integrative analysis of spatial transcriptomics data*, at lines

179-238). This section discusses analysis of the Visium spatial transcriptomics mouse brain dataset with H&E stain and is accompanied by Figure 4, which contains the relevant panels that the reviewer refers to in the comment above. Specifically, in addition to spatial patterns analysis already described, we added two analyses that leverage image analysis methods: summary statistics of image channels (Figure 4h) and nuclei segmentation of the H&Estain (Figure 4i,j).

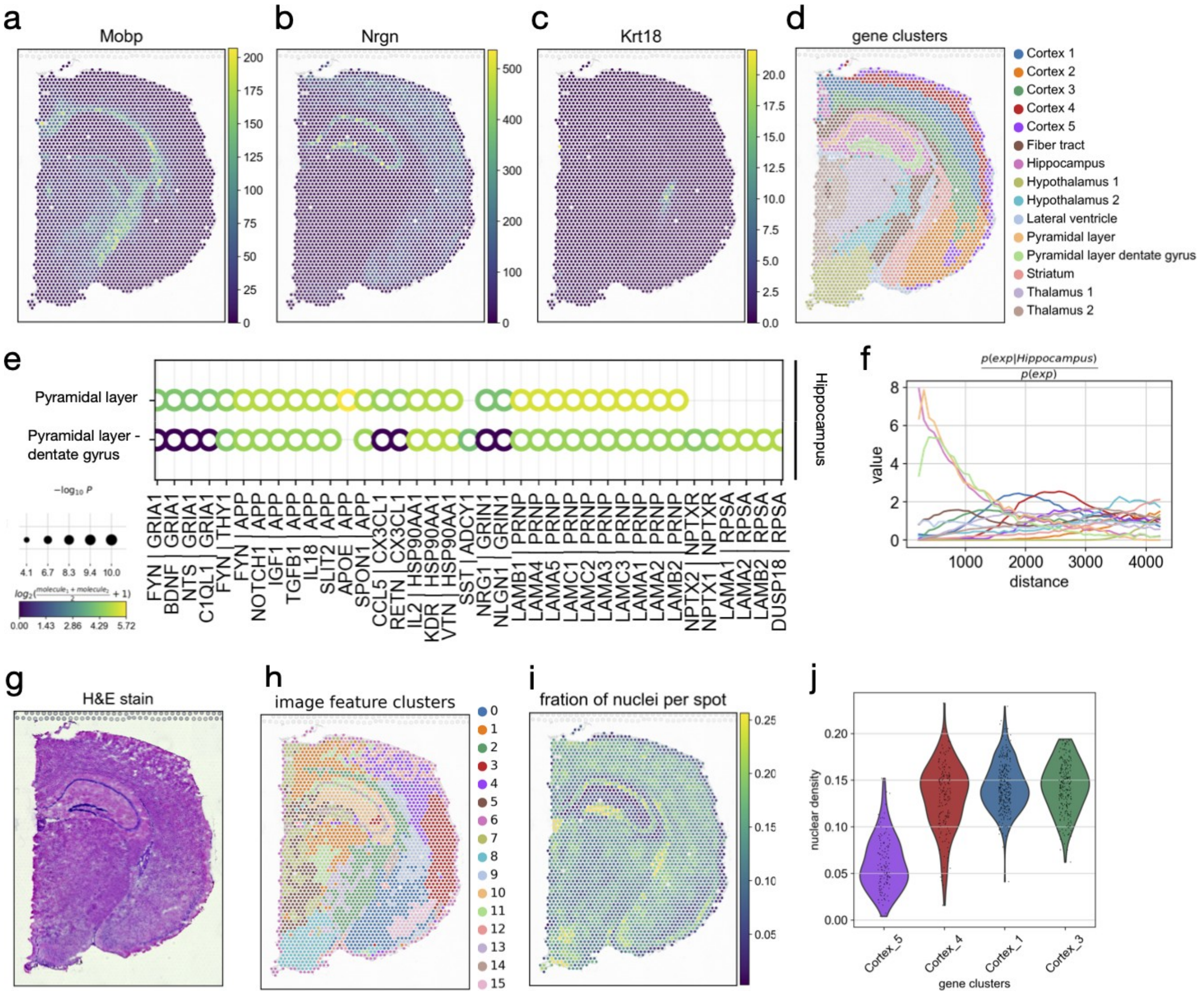

Figure 4: Analysis of mouse brain Visium dataset using Squidpy

(a) and (b) Gene expression in spatial context of two spatially variable genes (Mobp and Nrgn) as identified by Moran's I spatial autocorrelation statistic.

(c) Gene expression in spatial context of one spatially variable gene (*Krt18*) identified by Sepal method[12].

(d) Clustering of gene expression data plotted on spatial coordinates.

(e) Ligand-receptor interactions from the cluster “Hippocampus” to clusters “Pyramidal Layer” and “Pyramidal layer dentate gyrus”. Shown are a subset of significant ligand-receptor pairs queried using Omnipath database. Shown ligand-receptor pairs were filtered for visualization purposes, based on expression (mean expression >

13) and significant after FDR correction ($p < 0.01$).

(f) Co-occurrence score between "Hippocampus" and rest of the clusters. As seen qualitatively by clusters in spatial context in (d), “Pyramidal Layer” and “Pyramidal layer dentate gyrus” co-occur with the Hippocampus at short distances, given their proximity.

(g) H&E stain.

(h) Fraction of nuclei per Visium spot, computed using the cell segmentation algorithm StarDist[8].

(i) Clustering of summary image features (channel intensity mean, standard deviation, and 0.1, 0.5, 0.9th quantiles) derived from the H&E stain at each spot location (for quantitative comparison to gene clusters from (d) see Supplementary Fig. 2e)

(l) Violin plot of fraction of nuclei per Visium spot (g) for the cortical clusters (d).
This example is part of the Squidpy documentation (see https://squidpy.readthedocs.io/en/latest/auto_tutorials/tutorial_visium_hne.html)

To briefly summarize, the H&E feature clusters recapitulate areas of similar mean and standard deviation of image intensities. Therefore, areas like the hippocampus, that have overlapping image and gene clusters, can be characterised as having homogenous gene expression and morphology. Areas like the cortex, on the other hand, seem to have heterogeneous image features within one gene cluster. While the field may know this already to some extent in the cortex, in other novel imaging situations such as particular tumor entities this may be less clear and is in any case important to demonstrate quantitatively, in particular when wanting to test across conditions. In the manuscript, this part of the new section reads as follows (lines 209-238):

Squidpy’s feature extraction pipeline enables direct comparison and joint analysis of image and omics data. For instance, on the same Mouse brain coronal section data, we compared clusters computed from gene expression profiles with clusters computed from summary statistics (mean, standard deviation, 0.1, 0.5, and 0.9th quantiles) of the high-resolution H&E image channels (Fig. 4g,h). The image-based clusters recapitulate regions of image intensities with similar mean and standard variation, whereas the gene-based clusters are related to broad cell type definition. We can see that several image-based clusters are highly overlapping with the gene-based clusters, especially in the “Hippocampus” (54% overlap with image feature cluster 10), and the “Hypothalamus” (72% overlap with image feature cluster 8). This shows how members of such clusters share a similar definition both at morphology and molecular level which allows further characterization of the cluster. In contrast, the image-based clusters provide a different view of the data in the cortex (no overlap >33% with any image feature clusters) (Supplementary Fig. 3e). Here, gene clusters identify broad cortical layers whereas the image-based clusters separate different regions of the cortex based on changing local image intensities, indicating changes in cell density, morphology, or changes in the staining that are not captured by the gene expression data. For furtherexamination of these image feature clusters, we calculated a nuclei segmentation using StarDist[8] and extracted the number of nuclei per Visium spot (Fig. 4i). This nuclear count shows that image-based cluster 15 highlightsan area in the bottom part of the cortex with low cell density that is not covered fully by the gene cluster “Cortex 5”. In addition to explaining variation in the image-based clusters, the fraction of nuclei was combined with gene clusters to show that the nuclear density varies between the different cortical clusters (Fig. 4j). This indicates that gene expression clusters represent a different grouping of the cortex than the one identified by the image-based clustering. Such regions of different nuclear densities and morphology in the brain are of broad interest to neuroscientists[13,14]. Therefore, nuclear density and morphological

information represent valuable information to disentangle sources of variation in spatial transcriptomics data, and allow scientists to generate additional insights for the biological system of interest. In conclusion, we showed how Squidpy's integrative analysis workflows can fully leverage the spatial context and large microscopy image to generate novel hypothesisclasses in spatial transcriptomics data.

We believe that this extended discussion of the H&E feature clusters now addresses the reviewer's questions, and improves interpretability of this analysis. Finally, we would like to acknowledge that stLearn does not provide functionality to perform image segmentation (as stated in the comment) and therefore this analysis could have been replicated only partially.

2) A central claim of the manuscript is that Squidpy is technology-agnostic, and in support of this claim the authors demonstrate their approach across four different technologies: Visium, SeqFISH, 4i, and Imaging Mass Cytometry. This is a thoughtful and impressive demonstration which shows Squidpy can be used with spatial technologies which are spot-based and molecular imaging-based. However, the discussion of these modalities is incomplete, with the authors failing to mention higher-resolution bead-array modalities for spatial transcriptomics (e.g. HDST, Slide-Seq) and thick-sample 3D modalities for molecular imaging (e.g. merFISH, ISS, STARmap). The manuscript would greatly benefit from a considered and careful discussion of these modalities, and the manuscript should offer greater evidence supporting the claim of technology agnosticism by including these modalities or else significantly moderate its claims (e.g. it is not obvious from the manuscript Squidpy is capable of working with, say, 3D data).

We would like to thank the reviewer for these additional suggestions. To further strengthen the claim that Squidpyis technology-agnostic, we now added analysis examples for using Slide-SeqV2 (Figure 2h,k), and MERFISH (Figure 2d,e), to cover the classes of spatial modalities that the reviewer mentions above, and in addition a Mibi-tof dataset (Figure 3f), to showcase Squidpy's functionality to work with z-stacks data as well as different type of image data (single cell proteomics). All the aforementioned datasets are accompanied with separate extensive tutorials (https://squidpy.readthedocs.io/en/latest/tutorials.html) that showcase analysis that can be performed with Squidpy. We would also like to point out that we provide a tutorial on how to import any type of spatial data into the Squidpy/Scanpy infrastructure: https://squidpy.readthedocs.io/en/latest/auto_tutorials/tutorial_read_spatial.html.

We would like to thank the reviewer for pointing out that it was not obvious whether Squidpy is capable of workingwith 3D data. Squidpy was already capable of working with 3D spatial coordinates for e.g., spatial graph construction and visualisation, and to showcase this we added the MERFISH dataset (Figure 2d,e). Furthermore,we have now extended the Image Container to allow representation of images with x,y,z dimensions. With this extension, e.g., consecutive images of the same tissue can be represented in the same object, analyzed and interactively visualised with Napari. We extended the introduction to point this out (at lines 55-57):
They [graph and image data representations] are also able to deal with both 2D and 3D information, thus laying the foundations for large-scale molecular maps of tissues and organs.

In addition, we added a tutorial on how to use the Image Container with multiple z dimensions, using the Mibi-tof dataset as example: https://squidpy.readthedocs.io/en/latest/auto_tutorials/tutorial_mibitof.html and https://squidpy.readthedocs.io/en/latest/auto_tutorials/tutorial_image_container.html . The analyses performed in the tutorial are also used to describe the ImageContainer functionality in Figure 3.

In conclusion, the revised manuscript shows that Squidpy is a technology-agnostic framework that allows to leverage 2D and 3D spatial coordinates by showing examples on seven different spatial technologies.

3) The authors explicitly position their software package as an alternative to the existing Giotto package. The authors of Giotto demonstrated their package across nine different technologies versus Squidpy's four, including the bead (Slideseq) and 3D (merFISH, STARmap) modalities discussed above. Palla et al. benchmark Squidpy against Giotto, showing a claimed 10-fold speed-up and concluding "Squidpy outperforms similar functions provided by the Giotto toolkit, for any dataset and task" (Fig S1). This speedup is an outstanding achievement of which the authors should be very proud. However, as discussed above, it is not clear to me that Squidpy is compatible with all of the data modalities analyzed by Giotto -- e.g. what is the benchmark for dataset = "STARmap 3D visual cortex"? The manuscript would greatly benefit from a fair and nuanced comparison of these two packages across all relevant modalities/datasets, with appropriate evidence provided when making such strong claims; otherwise these claims should be significantly moderated.

We thank the reviewer for the useful comment and suggestion. In order to provide a more thorough comparisonwith Giotto, we have added the following points:

- An additional table (Supplementary Tab. 2) where functions in Giotto or Squidpy that specifically leverages the additional modalities of spatial omics data (coordinates and image) and tailored to a specific task, are listed. In the caption to Supplementary Tab. 2 we now compare Giotto and Squidpy as follows:

Supplementary Table 2. Comparison of available methods between Giotto and Squidpy. Comprehensive comparison between methods provided by the Giotto package and Squidpy, grouped byanalysis tasks. Implementation details between functions may differ but are deemed to perform the same analysis. The two frameworks are largely similar with respect to general tasks such as visualization. Giotto has a richer set of functions for the spatial graph but does not provide any methods to process and analyze the large tissue image, a modality often present in spatial omics data.

- We increased the number of datasets for which we performed the graph construction and neighborhood enrichment benchmark from four to seven datasets (Supplementary Fig. 1a). This comparison now also includes a MERFISH example, which is qualitatively similar to Starmap data, showing a rough 10x speedup on 3D spatial graph building and neighborhood enrichment:

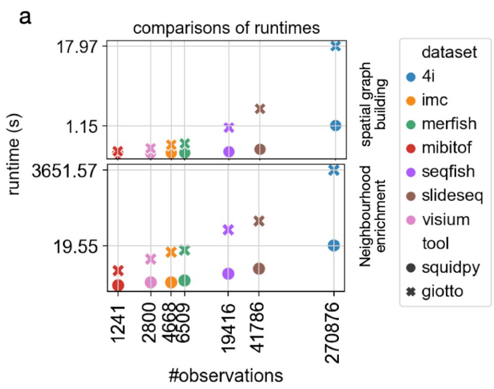

(a) Execution times for spatial graph building and neighborhood enrichment analysis, comparing seven spatial datasets at increasing number of observations. Squidpy outperforms similar functions provided by the Giotto toolkit[15]. In particular, reporting a minimum of 12-fold and a maximum of 15-fold speedup for the graph construction step, and a minimum of 8-fold and a maximum of 187-fold speedup for the neighborhood enrichment step. Reported are mean values for 10 runs, except for the 4i, SlideseqV2 and Seqfish neighbor enrichment test that was run only once in Giotto due to computational time demands. Axes are in log10 scale.

- We added a comparison of runtimes for the ligand-receptor interaction analysis (Supplementary Fig. 1b) to show Squidpy's faster runtime for this task:

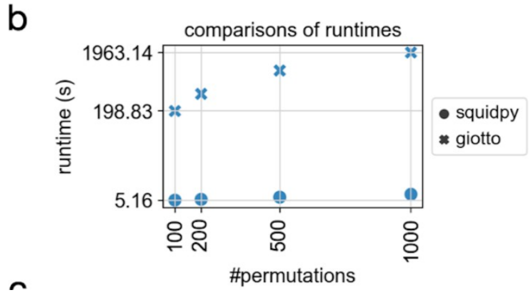

(b) Execution time for permutation test of ligand-receptor interaction analysis for one dataset (2800 observations, 15 clusters, 599 ligand-receptor pairs) at increasing number of permutations, computed using 1 thread (although Squidpy could parallelize easily, see (d)). Squidpy shows almost constant execution time (<7 seconds for 1000 permutations) compared to exponential increase for Giotto. It should be noted that the ligand-receptor pairs were the ones provided by Giotto ("mouse_ligand_receptors.txt" file), and 600 pairs were found in the dataset. Squidpy interface with Omnipath is able to retrieve 9014 interactions for the same dataset (data not shown), which correspondsto a 15X increase in available annotations. Axis is in log10 scale.

In conclusion, we moderated the claims in comparison to Giotto, acknowledging the rich set of analysis tools provided by the method (see caption Supplementary Tab. 2 above). We also removed the statement "for any dataset and task" (see caption Supplementary Fig. 1a above). Together with the extended usage-examples

showing now seven different spatial technologies, we believe that we now show that Squidpy compares favourably to existing methods and provide a fair comparison to Giotto.

Suggestions: 1) show a clear use case and compelling biological demonstration for spatial graphs + images, 2) show Squidpy is applicable to a wider range of techniques or significantly moderate claims 3) carefully re-work sections of the manuscript to make a more a nuanced and fair comparison to existing methods.

We thank the reviewer for the useful suggestions. In summary:

1. We reworked the manuscript to give a more extensive discussion of the power of Squidpy's integrative analysis by adding a new paragraph in the results section (titled: *Squidpy's workflow enables the integrative analysis of spatial transcriptomics data*, lines 179-238) and an additional Figure 4. We focused on one specific dataset (10x genomics Visium H&E mouse brain coronal section) and highlighted how integrating spatial, molecular and morphological information can enhance interpretabilityof spatial omics data.
2. We have now increased the number of datasets showcased (both in the paper and in the tutorials) to 7 (SeqFISH, MERFISH, Slide-seqV2, Visium, Mibi-tof, 4i, IMC data). We excluded other suggested datasets for the following reasons:
    a. HDST and ISS are closely related to SlideseqV2 and MERFISH/seqFISH (respectively) and therefore we are convinced that the addition does not provide additional insights on the broad applicability of Squidpy to spatial omics data analysis.
    b. Starmap is not openly available, but requires access at the website https://www.wangxiaolab.org/data-portal . We requested access but never received a reply. Nevertheless, Starmap is qualitatively similar to the MERFISH example and therefore it would not enhance the showcasing of Squidpy functionality.
3. We have qualitatively expanded the comparison to Giotto (Supplementary Table 2, Supplementary Fig 1) and added TissUUmaps (Supplementary Table 1, as suggested by Reviewer#1). We quantitatively compared Giotto's spatial graph construction, neighborhood enrichment analysis and ligand-receptor interaction function to Squidpy, finding a rough 10x speedup, thus in practice allowing Squidpy to be applied to larger data sets.

Minor comments:

1) Fig 2a, shows “lateral plate mesoderm” is co-enriched with "allantois" and "intermediate mesoderm", and “endothelium’ is enriched with “haematoendothelial progenitors”, and the authors point out this matches the results of Lohoff et al. However, sticking with this example, “haematoendothelial progenitors” are co-enriched with "allantois", "intermediate mesoderm", and "lateral plate mesoderm" in Lohoff et al, and this result is not evident in the analysis of Palla et al. While it seems by visual inspection that there are many commonalities between these two sets of results, it would inspire greater confidence if the manuscript included a more systematic comparison of these two sets of results, with explanations provided for discrepancies arising, and rationales given for why these

specific comparisons were drawn while others were omitted.

We thank the reviewer for the comment, we have rephrased the description in the main text (lines 90-93), changed the colormap for more intuitive comparisons with the Lohoff et al. analysis as well as provided motivation for our choice of visualized clusters in Fig 2c.

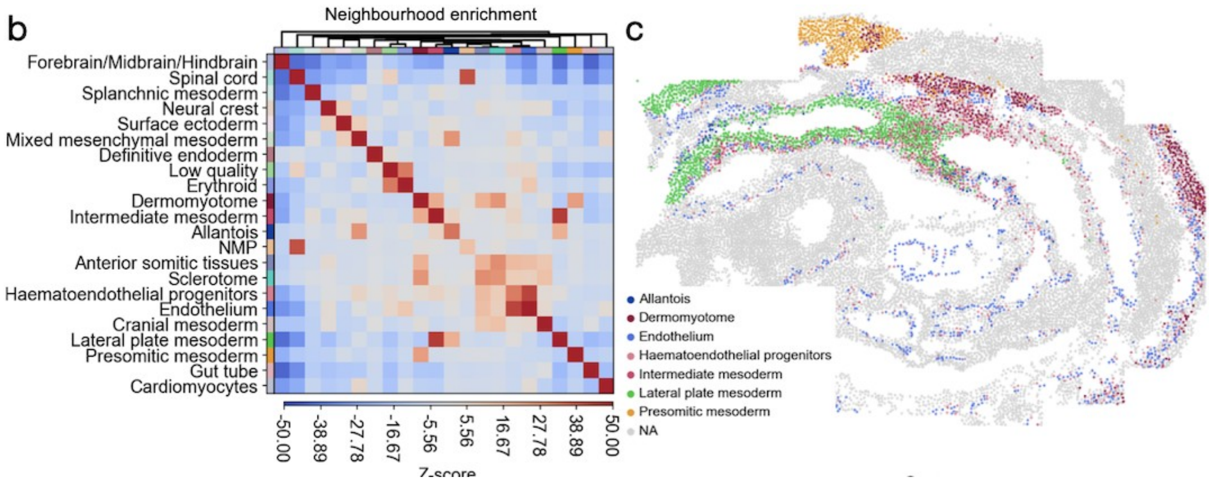

(b) Neighborhood enrichment analysis (1k permutations, see online methods) between cell clusters in spatial coordinates (spatial graph built with Delaunay triangulation method). Positive enrichment is found for the following cluster pairs: "Lateral plate mesoderm" with "Allantois" and "Intermediate mesoderm" clusters, "Endothelium" with "Haematoendothelial progenitors", "Anterior somitic tissues", "Sclerotome" and "Cranial mesoderm" clusters, "NMP" with "Spinal cord","Allantois" with "Mixed mesenchymal mesoderm", "Erythroid" with "Low quality", "Presomitic mesoderm" with "Dermomyotome", "Cardiomyocytes" with "Mixed mesenchymal mesoderm". All of these results were also reported by the original authors. Qualitative differences between analyses could arise due to the number of permutations employed (ours: 1000 permutations, original authors: 500 permutations) and construction of spatial graphs (ours: Delaunay triangulation, original authors: custom method leveraging segmentation masks).
(c) Visualization of selected clusters of the seqFISH mouse gastrulation dataset. The neighborhood enrichment score retrieves interactions for annotations both clustered ("lateral plate mesoderm" with "Allantois" and "Intermediate mesoderm" but also dispersed ("Haemathoendothelial progenitors" and "Endothelium") across the tissue area.

Unfortunately, we can only evaluate the similarities qualitatively, but we believe that it provides a sufficient level of confidence. We expanded the caption to Fig. 2b with the following statement:

Qualitative differences between analyses could arise due to the number of permutations employed (ours: 1000 permutations, original authors: 500 permutations) and construction of spatial graphs (ours: Delaunay triangulation, original authors: custom method leveraging segmentation masks).

2) Although the claimed ~10-fold speedup vs. Giotto is, as mentioned, quite impressive, visual inspection of Fig S1 suggests that this might be somewhat overstated, with only the 4i dataset definitively showing a speedup of this magnitude by visual inspection. The authors may want to annotate this chart to provide more obvious evidence for their claim. It is also unclear whether this large speedup applies across the full suite of functionality in Giotto (e.g. visualization) or specifically to graph-building and neighborhood enrichment only.

We thank the reviewer for the comment. We acknowledge that it is not a precise estimate and we therefore rephrased the statement in caption of Supplementary Fig. 1 to:

In particular, reporting a minimum of 12-fold and a maximum of 15-fold speedup for the graph construction step, and a minimum of 8-fold and a maximum of 187-fold speedup for the neighborhood enrichment step.

The full table of runtimes can be found here: https://github.com/theislab/squidpy_reproducibility/blob/master/notebooks/supp_figures/benchmarks/benchmark_graph.ipynb .
We additionally compared the functions for computing a ligand-receptor interaction permutation-based test (Supplementary Fig. 1b) at increased number of permutations (critical to reach a robust significance level). We also noted in the caption of Supplementary Fig. 1 that Giotto provides far fewer ligand-receptor annotations for this specific dataset. For the sake of a fair comparison, we could only compare these three functions with Giotto because the statistics and implementation closely resembled the ones in Squidpy (see Supplementary Table 2 fora list of functions available in the two methods).
As detailed in the caption of Supplementary Fig. 1, the mentioned speedup vs. Giotto refers to the graph construction and neighborhood enrichment. Comparing the efficiency of visualisation tools between Giotto and Squidpy would not be very informative: For static visualization, they both employ standard plotting libraries in the respective platforms. For interactive visualization, Giotto employs a custom viewer based on imagemagick/C whereas Squidpy employs Napari, an interactive image viewer in plain Python.
In both cases, large microscopy tissue images such as TIFF images acquired with Visium could be loaded.

3) It would be helpful if the authors can describe how they pre-process their datasets in the methods section.
Thank you for the suggestion. We added a section for dataset pre-processing (Section 4, Dataset pre-processing) in the methods that details the pre-processing that was applied to the datasets prior to the inclusion in the *squidpy.datasets* module. In addition, all code to reproduce the pre-processing (and the subsequent analysis) can be found in the reproducibility repository: https://github.com/theislab/squidpy_reproducibility, as also mentioned in the “Code and data availability” section.

4) Spelling error in Fig 2g (‘nucleous’)
Thank you for catching this typo. It is now corrected in the figure (‘nucleous’ -> ‘nucleus’).

References

1. Avila Cobos, F., Alquicira-Hernandez, J., Powell, J. E., Mestdagh, P. & De Preter, K. Benchmarking of cell type deconvolution pipelines for transcriptomics data. *Nat. Commun.* **11**, 5650 (2020).
2. Elosua, M., Nieto, P., Mereu, E., Gut, I. & Heyn, H. SPOTlight: Seeded NMF regression to Deconvolute Spatial Transcriptomics Spots with Single-Cell Transcriptomes. *bioRxiv* 2020.06.03.131334 (2020) doi:10.1101/2020.06.03.131334.
3. Dong, R. & Yuan, G.-C. SpatialDWLS: accurate deconvolution of spatial transcriptomic data. *Cold Spring Harbor Laboratory* 2021.02.02.429429 (2021) doi:10.1101/2021.02.02.429429.
4. Su, J. & Song, Q. DSTG: Deconvoluting Spatial Transcriptomics Data through Graph-based Artificial Intelligence. *Cold Spring Harbor Laboratory* 2020.10.20.347195 (2020) doi:10.1101/2020.10.20.347195.
5. Cable, D. M. *et al.* Robust decomposition of cell type mixtures in spatial transcriptomics. *Cold Spring Harbor Laboratory* 2020.05.07.082750 (2020) doi:10.1101/2020.05.07.082750.
6. Righelli, D. *et al.* SpatialExperiment: infrastructure for spatially resolved transcriptomics data in R using Bioconductor. *Cold Spring Harbor Laboratory* 2021.01.27.428431 (2021) doi:10.1101/2021.01.27.428431.
7. Stringer, C., Wang, T., Michaelos, M. & Pachitariu, M. Cellpose: a generalist algorithm for cellular segmentation. *Nat. Methods* **18**, 100–106 (2021).
8. Schmidt, U., Weigert, M., Broaddus, C. & Myers, G. Cell Detection with Star-convex Polygons. *arXiv [cs.CV]* (2018).
9. McQuin, C. *et al.* CellProfiler 3.0: Next-generation image processing for biology. *PLoS Biol.* **16**, e2005970 (2018).
10. Asp, M. *et al.* A Spatiotemporal Organ-Wide Gene Expression and Cell Atlas of the Developing

Human Heart. *Cell* **179**, 1647–1660.e19 (2019).

11. Dask Development Team. Dask: Library for dynamic task scheduling. (2016).
12. Anderson, A. & Lundeberg, J. sepal: Identifying Transcript Profiles with Spatial Patterns by Diffusion-based Modeling. *Bioinformatics* (2021) doi:10.1093/bioinformatics/btab164.
13. Amunts, K., Mohlberg, H., Bludau, S. & Zilles, K. Julich-Brain: A 3D probabilistic atlas of the human brain's cytoarchitecture. *Science* **369**, 988–992 (2020).
14. Ortiz, C., Carlén, M. & Meletis, K. Spatial Transcriptomics: Molecular Maps of the Mammalian Brain. *Annu. Rev. Neurosci.* (2021) doi:10.1146/annurev-neuro-100520-082639.
15. Dries, R. *et al.* Giotto, a pipeline for integrative analysis and visualization of single-cell spatial transcriptomic data. *bioRxiv* 701680 (2019) doi:10.1101/701680.

Decision Letter, first revision:

Subject: Decision on Nature Methods submission NMETH-BC45303A
Message: 27th Jul 2021

Dear Professor Theis,

Thank you for your letter detailing how you would respond to the reviewer concerns regarding your Brief Communication, "Squidpy: a scalable framework for spatial omics analysis". We have decided to invite you to revise your manuscript as you have outlined, before we reach a final decision on publication.

Please do not hesitate to contact me if you have any questions or would like to discuss these revisions further.

When revising your paper:

* include a point-by-point response to the reviewers and to any editorial suggestions

* please underline/highlight any additions to the text or areas with other significant changes to facilitate review of the revised manuscript

* address the points listed described below to conform to our open science requirements

* ensure it complies with our general format requirements as set out in our guide to authors at www.nature.com/naturemethods

* resubmit all the necessary files electronically by using the link below to access your home page

[REDACTED]

Note: This URL links to your confidential home page and associated information about manuscripts you may have submitted, or that you are reviewing for us. If you wish to forward this email to co-authors, please delete the link to your homepage.

We hope to receive your revised paper within 2 weeks. We are very aware of the difficulties caused by the COVID-19 pandemic to the community. If you cannot send it within this time, please let us know. In this event, we will still be happy to reconsider your paper at a later date so long as nothing similar has been accepted for publication at Nature Methods or published elsewhere.

OPEN SCIENCE REQUIREMENTS

REPORTING SUMMARY AND EDITORIAL POLICY CHECKLISTS
When revising your manuscript, please update your reporting summary and editorial policy checklists.

Reporting summary: https://www.nature.com/documents/nr-reporting-summary.zip
Editorial policy checklist: https://www.nature.com/documents/nr-editorial-policy-checklist.zip

If your paper includes custom software, we also ask you to complete a supplemental reporting summary.

Software supplement: https://www.nature.com/documents/nr-software-policy.pdf

Please submit these with your revised manuscript. They will be available to reviewers to aid in their evaluation if the paper is re-reviewed. If you have any questions about the checklist, please see http://www.nature.com/authors/policies/availability.html or contact me.

Please note that these forms are dynamic 'smart pdfs' and must therefore be downloaded and completed in Adobe Reader. We will then flatten them for ease of use by the reviewers. If you would like to reference the guidance text as you complete the template, please access these flattened versions at http://www.nature.com/authors/policies/availability.html.

IMAGE INTEGRITY
When submitting the revised version of your manuscript, please pay close attention to Digital Image Integrity Guidelines and to the following points below:

-- that unprocessed scans are clearly labelled and match the gels and western blots presented in figures.
-- that control panels for gels and western blots are appropriately described as loading on sample processing controls
-- all images in the paper are checked for duplication of panels and for splicing of gel lanes.

Finally, please ensure that you retain unprocessed data and metadata files after publication, ideally archiving data in perpetuity, as these may be requested during the peer review and production process or after publication if any issues arise.

DATA AVAILABILITY
We strongly encourage you to deposit all new data associated with the paper in a persistent repository where they can be freely and enduringly accessed. We recommend submitting the data to discipline-specific and community-recognized repositories; a list of repositories is provided here: http://www.nature.com/sdata/policies/repositories

All novel DNA and RNA sequencing data, protein sequences, genetic polymorphisms, linked genotype and phenotype data, gene expression data, macromolecular structures, and proteomics data must be deposited in a publicly accessible database, and accession codes and associated hyperlinks must be provided in the "Data Availability" section.

Refer to our data policies here: https://www.nature.com/nature-research/editorial-policies/reporting-standards#availability-of-data

To further increase transparency, we encourage you to provide, in tabular form, the data underlying the graphical representations used in your figures. This is in addition to our data-deposition policy for specific types of experiments and large datasets. For readers, the source data will be made accessible directly from the figure legend. Spreadsheets can be submitted in .xls, .xlsx or .csv formats. Only one (1) file per figure is permitted: thus if there is a multi-paneled figure the source data for each panel should be clearly labeled in the csv/Excel file; alternately the data for a figure can be included in multiple, clearly labeled sheets in an Excel file. File sizes of up to 30 MB are permitted. When submitting source

data files with your manuscript please select the Source Data file type and use the Title field in the File Description tab to indicate which figure the source data pertains to.

Please include a “Data availability” subsection in the Online Methods. This section should inform readers about the availability of the data used to support the conclusions of your study, including accession codes to public repositories, references to source data that may be published alongside the paper, unique identifiers such as URLs to data repository entries, or data set DOIs, and any other statement about data availability. At a minimum, you should include the following statement: “The data that support the findings of this study are available from the corresponding author upon request”, describing which data is available upon request and mentioning any restrictions on availability. If DOIs are provided, please include these in the Reference list (authors, title, publisher (repository name), identifier, year). For more guidance on how to write this section please see: http://www.nature.com/authors/policies/data/data-availability-statements-data-citations.pdf

CODE AVAILABILITY
Please include a “Code Availability” subsection in the Online Methods which details how your custom code is made available. Only in rare cases (where code is not central to the main conclusions of the paper) is the statement “available upon request” allowed (and reasons should be specified).

We request that you deposit code in a DOI-minting repository such as Zenodo, Gigantum or Code Ocean and cite the DOI in the Reference list. We also request that you use code versioning and provide a license.

For more information on our code sharing policy and requirements, please see: https://www.nature.com/nature-research/editorial-policies/reporting-standards#availability-of-computer-code

MATERIALS AVAILABILITY
As a condition of publication in Nature Methods, authors are required to make unique materials promptly available to others without undue qualifications.

Authors reporting new chemical compounds must provide chemical structure, synthesis and characterization details. Authors reporting mutant strains and cell lines are strongly encouraged to use established public repositories.

More details about our materials availability policy can be found at https://www.nature.com/nature-portfolio/editorial-policies/reporting-standards#availability-of-materials

ORCID

Nature Methods is committed to improving transparency in authorship. As part of our efforts in this direction, we are now requesting that all authors identified as ‘corresponding author’ on published papers create and link their Open Researcher and Contributor Identifier (ORCID) with their account on the Manuscript Tracking System (MTS), prior to acceptance. This applies to primary research papers only. ORCID helps the scientific community achieve unambiguous attribution of all scholarly contributions. You can create and link your ORCID from the home page of the MTS by clicking on ‘Modify my Springer Nature account’. For more information please visit please www.springernature.com/orcid.

Please do not hesitate to contact me if you have any questions or would like to discuss these revisions further. We look forward to seeing the revised manuscript and thank you for the opportunity to consider your work.

Sincerely,

Lin

Lin Tang, PhD
Senior Editor
Nature Methods

Reviewers' Comments:

Reviewer #1:
Remarks to the Author:
I am very pleased with the revisions. No further comments.

Reviewer #2:
Remarks to the Author:
The authors have substantially re-structured the manuscript, making both technical advancements and general workflow clear and easy to grasp. A great merit of the paper is the collection of examples, including both full pipelines and shorter examples, making it a fantastic source for anyone (also with limited python experience) for analysis of a broad range of spatial comics types.

The authors have also responded very well to all previous critiques and made several important improvements/additions to the Squidpy toolbox.

I therefore find the paper well worthy of publication.

Minor comment: figure texts 4 h and i do not match the content of the figure. Please either swap images or texts.

Reviewer #3:
Remarks to the Author:
The authors have made significant efforts to address the original comments, and present present a substantially improved paper. In response to our earlier major comments, they have (1) reworked the section on integrative image/ST analysis, (2) demonstrated Squidpy is extensible to 3D, and (3) substantially qualified their comparisons to Giotto.

We find the author's revisions (2) and (3) well executed and fully satisfactory, and have no further concerns in these areas.

We unfortunately do not yet find the authors' responses to our first major comment fully satisfactory, although they are on the right track. The authors seek to showcase their integrative image/ST analysis by characterizing variation in cell density between cortical gene clusters -- this is essentially a good idea. However, the specific analyses they have added to Figure 4 in support of this demo raise new concerns, listed below:

The authors now show in 4j that gene cluster Cortex_5 has significantly lower nuclear density than the other cortical gene clusters. By inspection, Cortex_5 appears to be the outermost cortical layer of the slice, and is only a single hex (Visium spot) thick in many places. Is it possible that the outer layer of hexes does not fully overlap the tissue, leading to a relative undercount of nuclei? Has this layer-specific density been observed elsewhere? The result is promising, but I would like to see more validation that this demonstration is not measuring a technical artifact.
The authors should include significance testing between the other gene clusters in 4j, to support their claim that nuclear density varies between clusters.
The authors may want to explain why Cortex_2 is excluded from the analysis.
The captions for (h) and (i) appear to be swapped.

In addition to these new concerns, we remain concerned that even with the new analyses, the authors have not fully addressed the original issue raised, which is that to the uninitiated, the integrated analysis appears unmotivated. However, the authors appear to be on the right track, and this can probably be

resolved through thoughtful re-writing rather than new analyses. For instance, can the authors make a stronger statement than “such regions of different nuclear densities and morphology in the brain are of broad interest to neuroscientists?” The references provided refer to detection of specific brain nuclei (areas) by variations in cell body density. Can the authors place their results in that context, if appropriate?

Altogether, we appreciate the authors efforts in these revisions. We hope the authors can resolve these remaining issues.

Author Rebuttal, first revision:

Point-by-point response to the reviewers' comments

Title: Squidpy: a scalable framework for spatial omics analysis

Authors: Giovanni Palla*1,2, Hannah Spitzer*1, Michal Klein1, David Fischer1,2, Anna Christina Schaar1,2, Louis Benedikt Kuemmerle1,4, Sergei Rybakov1,3, Ignacio L. Ibarra1, Olle Holmberg1, Isaac Virshup5, Mohammad Lotfollahi1,2, Sabrina Richter1,2, Fabian J. Theis1,2,3+

1 Institute of Computational Biology, Helmholtz Center Munich, Germany.
2 TUM School of Life Sciences Weihenstephan, Technical University of Munich, Germany.
3 Department of Mathematics, Technical University of Munich, Germany.
4 Institute for Tissue Engineering and Regenerative Medicine (iTERM), Helmholtz Center Munich, Germany
5 Department of Anatomy and Physiology, University of Melbourne, Australia.

*equal contribution
+Correspondence: fabian.theis@helmholtz-muenchen.de

In the following, we present our response to the reviewers comments. We give **comments (black)**, **point-by-point answers (green)** to the questions and in parts **copy parts of the text or specific panels (beige)**, which directly correspond to comments or reference to them.

Reviewer #1 (Remarks to the Author):

I am very pleased with the revisions. No further comments.

We are very happy that this revision was so well received.

Reviewer #2 (Remarks to the Author):

The authors have substantially re-structured the manuscript, making both technical advancements and general workflow clear and easy to grasp. A great merit of the paper is the collection of examples, including both full pipelines and shorter examples, making it a fantastic source for anyone (also with limited python experience) for analysis of a broad range of spatial comics types.

The authors have also responded very well to all previous critiques and made several important improvements/additions to the Squidpy toolbox.

I therefore find the paper well worthy of publication.

Minor comment: figure texts 4 h and i do not match the content of the figure. Please either swap images or texts.

We are grateful to the reviewer for the positive evaluation of our manuscript. We appreciate that our effort in making the package accessible, well documented and thoroughly tested have been highlighted.
We also want to thank the reviewer for spotting the caption error. It is now correct in the new version of the manuscript.

Reviewer #3 (Remarks to the Author):

The authors have made significant efforts to address the original comments, and present a substantially improved paper. In response to our earlier major comments, they have (1) reworked the section on integrative image/ST analysis, (2) demonstrated Squidpy is extensible to 3D, and (3) substantially qualified their comparisons to Giotto.

We find the author's revisions (2) and (3) well executed and fully satisfactory, and have no further concerns in these areas.

We unfortunately do not yet find the authors' responses to our first major comment fully satisfactory, although they are on the right track. The authors seek to showcase their integrative image/ST analysis by characterizing variation in cell density between cortical gene clusters -- this is essentially a good idea. However, the specific analyses they have added to Figure 4 in support of this demo raise new concerns, listed below:

We thank the reviewer for the valuable feedback and address the remaining concerns in a point-by-point response below.

The authors now show in 4j that gene cluster Cortex_5 has significantly lower nuclear density than the other cortical gene clusters. By inspection, Cortex_5 appears to be the outermost cortical layer of the slice, and is only a single hex (Visium spot) thick in many places. Is it possible that the outer layer of hexes does not fully overlap the tissue, leading to a relative undercount of nuclei? Has this layer-specific density been observed elsewhere? The result is promising, but I would like to see more validation that this demonstration is not measuring a technical artifact.
The authors should include significance testing between the other gene clusters in 4j, to support their claim that nuclear density varies between clusters.

We thank the reviewer for raising this issue. Indeed the cluster Cortext_5 lies at the outermost layer of the slice, and low nuclear density could be an issue of masking, so we now performed analysis to show this is not the case; at the same time of course demonstrating that Squidpy enables answering these types of questions easily.
First, we would like to point out that for the analysis shown in Figure 4, we explicitly use only the spots that were determined to overlap with the tissue by the standard SpaceRanger pipeline. This pipeline performs a spot-filtering step where only high-quality spots that overlap the tissue are retained. However, we understand that a more stringent threshold can be desirable, especially considering that our claim is specifically based on features that we are able to extract with Squidpy from the image alone.
Therefore, we set up to perform a robustness analysis, which we now describe in the new Supplementary Figure 6 and copied below. In short, we binarized the original image based on Otsu's thresholding method, and then filtered out spots according to multiple, increasing quantile thresholds on mean intensity value (1 = every pixel overlap with tissue, 0, no pixels overlap with tissue). We then re-computed differential testing of nuclei density between cluster "Cortex_5" and the other cortical layers for each of the thresholds. Despite increased filtering, cluster "Cortex_5" still retained a significant decrease in nuclei density compared to other cortical layers. Since we scanned a large range of filtering thresholds, this led us to conclude that this is not due to technical noise i.e. a masking/boundary effect.
Besides adding the new supplementary figure, we added a short sentence on this in the main text.

Furthermore, Squidpy image-processing tools allow to quickly validate the robustness of such findings, by refining the selection of spots that fully overlap the detected tissue area in order to remove potential false positives (Supplementary Fig. 6)

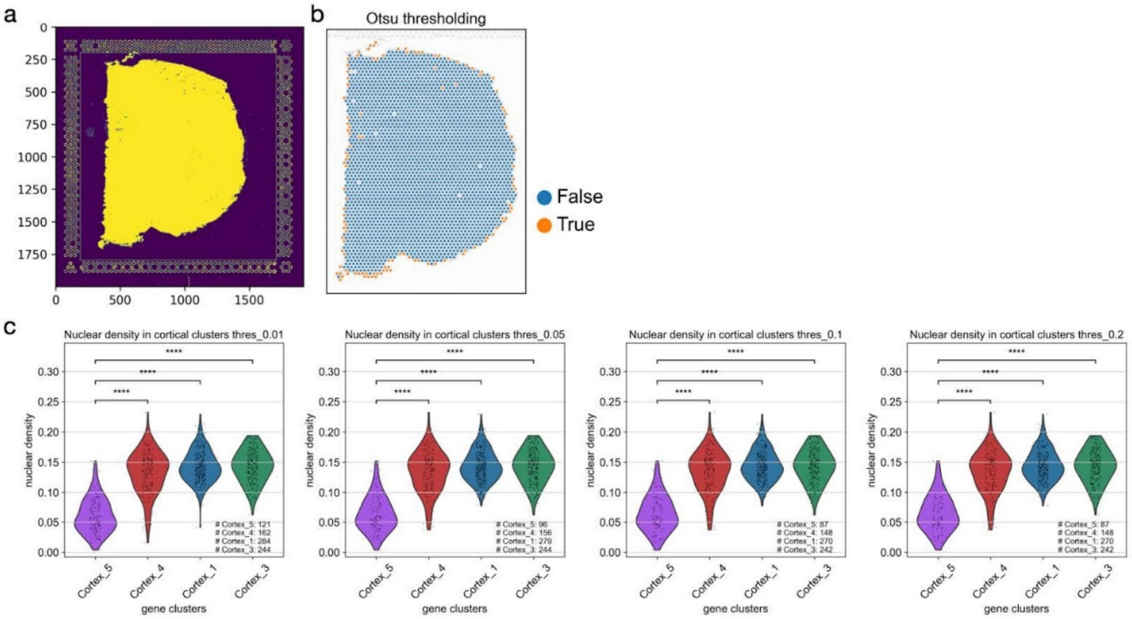

Supplementary Figure 6. Thresholding the tissue image to filter spots that do not fully overlap with detected tissue does not impact differential nuclei density between cortical layers.

(a) Visualization of Otsu's thresholding of Visium slide (detected tissue and fiducial spots). The resulting image is a binarized version based on the threshold selected by Otsu's method. Yellow pixels are 1 whereas blue pixels are 0.

(b) Spots filtered based on 0.05 quantile on Otsu's thresholding density. Orange spots are spots that are filtered by the threshold, since they do not retain a mean intensity value that is above the selected quantile (0.05). Blue spots are instead kept for downstream analysis, as they retain high overlap with the detected tissue. This shows that most of the removed spots are the ones at the boundary of the tissue, only partially overlapping with the detected tissue.

(c) Same analysis from Figure 4j, now performed at increasing quantile values for the Otsu's thresholding from (b). From left to right, nuclei density estimation is performed only including spots that are above the selected quantile value: 0.01, 0.05, 0.1, 0.2. Interestingly, despite the increased number of spots filtered out at increasing quantile values, the high-confidence spots of cluster "Cortex_5" still show a significant decrease in nuclei density as compared to the other cortical layers. In the figure annotation, absolute number of spots for each cluster is reported, notice that the absolute number decreases at increased quantile's thresholding value. The result without Otsu's thresholding is reported in the updated Figure 4.j.

Test performed is Mann-Whitney-Wilcoxon test two-sided with Bonferroni correction, p-value annotation legend is the following:

ns: 5.00e-02 < p <= 1.00e+00
*: 1.00e-02 < p <= 5.00e-02
**: 1.00e-03 < p <= 1.00e-02
***: 1.00e-04 < p <= 1.00e-03
****: p <= 1.00e-04

The authors may want to explain why Cortex_2 is excluded from the analysis.

Thank you for raising this. The reason we excluded cluster Cortex_2 is because it is located in the lower part of the cortex and largely overlaps with the cortical subplate (amygdalar nucleus). It is therefore part of the Cortex,

but not part of the isocortex, our region of interest in order to show the differential nuclei density score that we are able to compute with Squidpy. We added an additional, short explanation to the figure caption.

The cluster Cortex_2 was omitted from this analysis because it entails a different region of the cortex (cortical subplate) for which the differential nuclei density score between iso-cortical layers is not relevant.

The captions for (h) and (i) appear to be swapped.

Thanks, we swapped the caption text of Figure 4 in the manuscript

In addition to these new concerns, we remain concerned that even with the new analyses, the authors have not fully addressed the original issue raised, which is that to the uninitiated, the integrated analysis appears unmotivated. However, the authors appear to be on the right track, and this can probably be resolved through thoughtful re-writing rather than new analyses. For instance, can the authors make a stronger statement than "such regions of different nuclear densities and morphology in the brain are of broad interest to neuroscientists?" The references provided refer to detection of specific brain nuclei (areas) by variations in cell body density. Can the authors place their results in that context, if appropriate?

We agree that specifying use cases for integrated analysis will likely increase the value of the manuscript. In the previous revision, we restructured the manuscript and dedicated an entire new section and figure (Figure 4) to showcase how different Squidpy functionalities can be tied together for the integrative analysis of spatial transcriptomics data. The particular example highlighted (differential nuclear density in the outermost cortical layer) is an established findings of neuroanatomy, we added a citation to Kandel et al.[1] to support this claim.

This example is an instance for a conceptual link between imaging-based pathology, which typically yields a high-level description of an organ which is centred around tissue hallmarks, and the high-resolution information on gene expression available from spot transcriptomics. We argue that a toolbox for integrated analysis of gene expression and image data is necessary to facilitate discovery of patterns in the future and to improve knowledge transfer between Pathology and Molecular Biology. Future research based on our toolbox may inquire into positioning of vessels in the tissue, tumor-stroma boundaries, and similar sample characteristics that explain spatial variation which are obvious from the image modality.

We hope that with the reformulation now in the manuscript (lines 210-212, 246-251), the additional specification of better ways to interrogate uncertainties on effects depending eg on technical noise (see Supp Fig 6 as discussed above) and the established finding, we can now more clearly demonstrate the advantage for users to work in an integrated toolbox for both expression and spatial analysis.

Altogether, we appreciate the authors efforts in these revisions. We hope the authors can resolve these remaining issues.

We thank the reviewers for the useful suggestions, we hope we were able to address all the remaining issues in the updated version of the manuscript.

References

1. Kandel, E., Koester, J. D., Mack, S. H. & Siegelbaum, S. *Principles of Neural Science, Sixth Edition*. (McGraw-Hill Education, 2021).

Decision Letter, second revision:

Subject: AIP Decision on Manuscript NMETH-A45303B
Message:

Our ref: NMETH-A45303B

30th Aug 2021

Dear Dr. Theis,

Thank you for submitting your revised manuscript "Squidpy: a scalable framework for spatial omics analysis" (NMETH-A45303B). It has now been seen by Reviewer 3 and their comments are below. The reviewers find that the paper has improved in revision, and therefore we'll be happy in principle to publish it in Nature Methods, pending minor revisions to comply with our editorial and formatting guidelines.

We are now performing detailed checks on your paper and will send you a checklist detailing our editorial and formatting requirements in about a week. Please do not upload the final materials and make any revisions until you receive this additional information from us.

TRANSPARENT PEER REVIEW
Nature Methods offers a transparent peer review option for new original research manuscripts submitted from 17th February 2021. We encourage increased transparency in peer review by publishing the reviewer comments, author rebuttal letters and editorial decision letters if the authors agree. Such peer review material is made available as a supplementary peer review file. **Please state in the cover letter 'I wish to participate in transparent peer review' if you want to opt in, or 'I do not wish to participate in transparent peer review' if you don't.** Failure to state your preference will result in delays in accepting your manuscript for publication.
Please note: we allow redactions to authors' rebuttal and reviewer comments in the interest of confidentiality. If you are concerned about the release of confidential data, please let us know specifically what information you would like to have removed. Please note that we cannot incorporate redactions for any other reasons. Reviewer names will be published in the peer review files if the reviewer signed the comments to authors, or if reviewers explicitly agree to release their name. For more information, please refer to our FAQ page.

Thank you again for your interest in Nature Methods Please do not hesitate to contact me if you have any questions.

Sincerely,

Lin

Lin Tang, PhD
Senior Editor
Nature Methods

ORCID
IMPORTANT: Non-corresponding authors do not have to link their ORCIDs but are encouraged to do so. Please note that it will not be possible to add/modify ORCIDs at proof. Thus, please let your co-authors know that if they wish to have their ORCID added to the paper they must follow the procedure described in the following link prior to acceptance:
https://www.springernature.com/gp/researchers/orcid/orcid-for-nature-research

Reviewer #3 (Remarks to the Author):

The authors have addressed my comments.

Final Decision Letter:

Subject: Decision on Nature Methods submission NMETH-A45303C
Message:

21st Nov 2021

Dear Professor Theis,

I am pleased to inform you that your Article, "Squidpy: a scalable framework for spatial omics analysis", has now been accepted for publication in Nature Methods. Your paper is tentatively scheduled for publication in our February print issue, and will be published online prior to that. The received and accepted dates will be 19th Feb 2021 and 21st Nov 2021. This note is intended to let you know what to expect from us over the next month or so, and to let you know where to address any further questions.

In approximately 10 business days you will receive an email with a link to choose the appropriate publishing options for your paper and our Author Services team will be in touch regarding any additional information that may be required.

You will not receive your proofs until the publishing agreement has been received through our system.

Your paper will now be copyedited to ensure that it conforms to Nature Methods style. Once proofs are generated, they will be sent to you electronically and you will be asked to send a corrected version within 24 hours. It is extremely important that you let us know now whether you will be difficult to contact over the next month. If this is the case, we ask that you send us the contact information (email, phone and fax) of someone who will be able to check the proofs and deal with any last-minute problems.

If, when you receive your proof, you cannot meet the deadline, please inform us at rjsproduction@springernature.com immediately.

If you have any questions about our publishing options, costs, Open Access requirements, or our legal forms, please contact ASJournals@springernature.com

Once your manuscript is typeset and you have completed the appropriate grant of rights, you will receive a link to your electronic proof via email with a request to make any corrections within 48 hours. If, when you receive your proof, you cannot meet this deadline, please inform us at rjsproduction@springernature.com immediately.

Once your paper has been scheduled for online publication, the Nature press office will be in touch to confirm the details.

Content is published online weekly on Mondays and Thursdays, and the embargo is set at 16:00 London time (GMT)/11:00 am US Eastern time (EST) on the day of publication. If you need to know the exact publication date or when the news embargo will be lifted, please contact our press office after you have submitted your proof corrections. Now is the time to inform your Public Relations or Press Office about your paper, as they might be interested in promoting its publication. This will allow them time to prepare an accurate and satisfactory press release. Include your manuscript tracking number NMETH-A45303C and the name of the journal, which they will need when they contact our office.

About one week before your paper is published online, we shall be distributing a press release to news organizations worldwide, which may include details of your work. We are happy for your institution or funding agency to prepare its own press release, but it must mention the embargo date and Nature Methods. Our Press Office will contact you closer to the time of publication, but if you or your Press Office have any inquiries in the meantime, please contact press@nature.com.

If you are active on Twitter, please e-mail me your and your coauthors' Twitter handles so that we may

tag you when the paper is published.

Please note that *Nature Methods* is a Transformative Journal (TJ). Authors may publish their research with us through the traditional subscription access route or make their paper immediately open access through payment of an article-processing charge (APC). Authors will not be required to make a final decision about access to their article until it has been accepted. Find out more about Transformative Journals

Authors may need to take specific actions to achieve compliance with funder and institutional open access mandates. For submissions from January 2021, if your research is supported by a funder that requires immediate open access (e.g. according to Plan S principles) then you should select the gold OA route, and we will direct you to the compliant route where possible. For authors selecting the subscription publication route our standard licensing terms will need to be accepted, including our self-archiving policies. Those standard licensing terms will supersede any other terms that the author or any third party may assert apply to any version of the manuscript.

If you have posted a preprint on any preprint server, please ensure that the preprint details are updated with a publication reference, including the DOI and a URL to the published version of the article on the journal website.

To assist our authors in disseminating their research to the broader community, our SharedIt initiative provides you with a unique shareable link that will allow anyone (with or without a subscription) to read the published article. Recipients of the link with a subscription will also be able to download and print the PDF. As soon as your article is published, you will receive an automated email with your shareable link.

Please note that you and your coauthors may order reprints and single copies of the issue containing your article through Nature Research Group's reprint website, which is located at http://www.nature.com/reprints/author-reprints.html. If there are any questions about reprints please send an email to author-reprints@nature.com and someone will assist you.

Please feel free to contact me if you have questions about any of these points. Thank you very much again for publishing this work at Nature Methods!

Best regards,

Lin

Lin Tang, PhD

Senior Editor
Nature Methods

** Visit the Springer Nature Editorial and Publishing website at www.springernature.com/editorial-and-publishing-jobs for more information about our career opportunities. If you have any questions please click here.**
